# The SCF^Dia2^ Ubiquitin E3 Ligase Ubiquitylates Sir4 and Functions in Transcriptional Silencing

**DOI:** 10.1371/journal.pgen.1002846

**Published:** 2012-07-26

**Authors:** Rebecca J. Burgess, Hui Zhou, Junhong Han, Qing Li, Zhiguo Zhang

**Affiliations:** Department of Biochemistry and Molecular Biology, Mayo Clinic College of Medicine, Rochester, Minnesota, United States of America; Purdue University, United States of America

## Abstract

In budding yeast, transcriptional silencing, which is important to regulate gene expression and maintain genome integrity, requires silent information regulator (Sir) proteins. In addition, Rtt106, a histone chaperone involved in nucleosome assembly, functions in transcriptional silencing. However, how transcriptional silencing is regulated during mitotic cell division is not well understood. We show that cells lacking Dia2, a component of the SCF^Dia2^ E3 ubiquitin ligase involved in DNA replication, display defects in silencing at the telomere and *HMR* locus and that the F-box and C-terminal regions of Dia2, two regions important for Dia2's ubiquitylation activity, are required for proper transcriptional silencing at these loci. In addition, we show that Sir proteins are mislocalized in *dia2*Δ mutant cells. Mutations in Dia2 and Rtt106 result in a synergistic loss of silencing at the *HMR* locus and significant elevation of Sir4 proteins at the *HMR* locus, suggesting that silencing defects in *dia2*Δ mutant cells are due, at least in part, to the altered levels of Sir4 at silent chromatin. Supporting this idea, we show that SCF^Dia2^ ubiquitylates Sir4 *in vitro* and *in vivo*. Furthermore, Sir4 binding to silent chromatin is dynamically regulated during the cell cycle, and this regulation is lost in *dia2*Δ mutant cells. These results demonstrate that the SCF^Dia2^ complex is involved in transcriptional silencing, ubiquitylates Sir4, and regulates transcriptional silencing during the cell cycle.

## Author Summary

Heterochromatin is important for the maintenance of genome stability and regulation of gene expression. Heterochromatin protein 1 (HP1), a protein that binds to histone H3 methylated at lysine 9 (H3K9me3) at heterochromatin loci in mammalian cells, is dynamically regulated during the cell cycle by phosphorylation of histone H3 serine 10 (H3S10ph). Compared to mammalian cells, transcriptional silencing at budding yeast silent chromatin requires silent information regulator (Sir) proteins, and H3K9me3 and H3S10ph are not present in budding yeast. Therefore, it is not known whether and how silent chromatin in budding yeast is regulated during the cell cycle. Here, we show that the SCF^Dia2^ ubiquitin E3 ligase complex regulates transcriptional silencing. We show that SCF^Dia2^ ubiquitylates Sir4, a structural component of yeast silent chromatin, and that Sir4 levels decrease during the cell cycle in a Dia2-dependent manner. Concomitant with the reduction of Sir4 at telomeric silent chromatin during the cell cycle, the expression of a telomere-linked gene increases. Therefore, we propose that transcriptional silencing at budding yeast silent chromatin is regulated during the cell cycle, in part by SCF^Dia2^-mediated Sir4 ubiquitylation on chromatin.

## Introduction

Chromatin structure governs a host of cellular processes, including gene expression, DNA replication, and DNA repair [1]. In higher eukaryotic cells, chromatin is classified into two major forms, euchromatin and heterochromatin, based on cytological staining. Euchromatin, the less dense and gene-rich form of chromatin, is associated with active gene transcription, whereas heterochromatin has a more compact structure and is associated with gene silencing. Though poor in genes, heterochromatin is important for development, centromere formation and the maintenance of genome integrity [2]–[4]. Therefore, it is important to understand how heterochromatin is formed and inherited during S phase of the cell cycle.

The budding yeast, *Saccharomyces cerevisiae*, has three heterochromatin-like loci: telomeres, the *HM* cryptic mating type loci (*HMR* and *HML*) and ribosomal DNA (rDNA) repeats. The initiation and maintenance of silent chromatin in budding yeast require Silent Information Regulator (Sir) proteins. While silencing at telomeres and the *HM* loci is regulated by many of the same factors, including Sir2, Sir3 and Sir4, only Sir2 is required for rDNA silencing. A stepwise model has been proposed for silent chromatin formation [5], [6]. For instance, at the *HMR* locus, Sir1 and Sir4 are recruited to the E silencer, a DNA sequence element containing binding sites for the origin-recognition complex (ORC) and transcription factors Rap1 and Abf1, through protein-protein interactions. Sir4 then recruits Sir2, a NAD^+^-dependent histone deacetylase, which deacetylates lysine residues on histones H3 and H4, including histone H4 lysine 16 (H4K16). This leads to the recruitment and binding of Sir3 and Sir4 to the adjacent nucleosome, as Sir3 and Sir4 bind hypoacetylated histones with higher affinity. This cycle of histone deacetylation and Sir protein binding to hypoacetylated nucleosomes leads to the spread of Sir proteins across the entire silent chromatin domain [5]–[7]. Despite the fact that protein factors and histone modifications involved in silent chromatin formation and maintenance in budding yeast are different from those in mammalian cells, this mechanism of step-wise formation of silent chromatin is likely to be conserved in higher eukaryotic cells [5], [8]. Importantly, despite advances made in understanding chromatin structure and transcriptional silencing, how silent chromatin is inherited and maintained during S phase of the cell cycle remains elusive.

During S phase of the cell cycle, nucleosomes ahead of the replication fork are disassembled to facilitate access of DNA replication machinery to DNA. Immediately following DNA replication, replicated DNA is reassembled into nucleosomes using both newly-synthesized histones and parental histones in a process called DNA replication-coupled nucleosome assembly. It is known that deposition of newly-synthesized H3–H4 requires histone H3–H4 chaperones, including CAF-1, Asf1 and Rtt106 [9]. Various studies in budding yeast indicate that these histone chaperones function in two parallel pathways in transcriptional silencing: an Asf1 dependent pathway and a CAF-1 dependent pathway [10], [11]. For instance, *asf1*Δ or *rtt106*Δ cells exhibit reduced silencing at both telomeres and the *HM* loci when combined with mutations in Cac1, the large subunit of CAF-1 [10]–[13]. In addition, Sir proteins are mislocalized in cells lacking both Rtt106 and CAF-1 [11]. On the other hand, *rtt106Δ asf1*Δ double mutant cells do not exhibit synergistic silencing defects, suggesting that Rtt106 and Asf1 function in the same genetic pathway in transcriptional silencing [13]. These results support the idea that nucleosome assembly factors are important for proper formation and inheritance of silent chromatin structure in budding yeast [6].

Dia2 is an F-box containing protein that serves as the substrate recognition component of a SCF (Skp1/Cullin/F-box protein) ubiquitin E3 ligase. Cells lacking Dia2 exhibit gross chromosomal rearrangements and sensitivity to cytotoxic agents, indicative of a role for Dia2 in maintaining genome integrity [14]–[16]. Furthermore, Dia2 has been shown to be important during DNA replication [15]–[17]. The F-box domain in each of 11 known F-box containing proteins in budding yeast interacts with the SCF component Skp1, enabling interactions between the ubiquitylation machinery and substrate [18]. In addition to the F-box domain, Dia2 has two additional important domains: a tetratricopeptide repeat (TPR) domain at the N-terminus involved in mediating Dia2's interaction with replisome components [14], [17] and a leucine rich repeat (LRR) region at the C-terminus. The LRR domain in other F-box containing proteins is known to be involved in substrate binding [19].

In a genetic screen designed to identify genes that function in parallel to *RTT106* in silencing at the *HMR* locus, we discovered that *DIA2*, when deleted, enhances *rtt106*Δ silencing defects at the *HMR* locus. Structure-function studies revealed that the Dia2 F-box and LRR regions are important for transcriptional silencing. Furthermore, both Sir3 and Sir4 are mislocalized in *dia2*Δ cells, and Sir4 binding to the *HMR* locus is significantly elevated in *dia2Δ rtt106*Δ mutant cells. In addition, we show that Sir4 is ubiquitylated in yeast cells in a Dia2-dependent manner and that Sir4 levels on chromatin are cell cycle regulated, and this regulation is lost in *dia2*Δ mutant cells. Therefore, we suggest that the SCF^Dia2^ E3 ligase functions in transcriptional silencing, in part through the regulation of Sir4 ubiquitylation.

## Results

### SCF^Dia2^ functions in transcriptional silencing

We identified *RTT106* in a screen for genes that function in parallel with PCNA in silencing at the *HMR* silent mating type locus [13]. Using a similar approach, we set out to identify genes that functioned in parallel with *RTT106* in transcriptional silencing. Briefly, we used the synthetic genetic array (SGA) approach [20], [21] to combine the *rtt106*Δ single mutant containing the *HMR::GFP* reporter gene with each of ∼4700 yeast deletion mutants. The *HMR::GFP* reporter contains the green fluorescent protein (GFP) integrated at the *HMR* silent mating type locus within the *a1* gene; thus, GFP is silenced. Once double mutants containing the *HMR::GFP* reporter gene were selected, flow cytometry was used to identify those genes from the collection of mutants that when combined with *rtt106*Δ, resulted in a significant elevation in the percentage of GFP expressing cells.

We identified five genes (*CAC1*, *CAC2*, *SIR1*, *ARD1*, and *DIA2*) that enhanced the silencing defects of *rtt106*Δ cells. Cac1 and Cac2 are two subunits of the histone chaperone CAF-1, and Sir1 is necessary for initiation of silent chromatin formation at the *HM* loci [22], [23]. Deletion of *SIR1*, *CAC1*, or *CAC2* is known to enhance the silencing defects of *rtt106*Δ mutant cells [11], [13]. In addition, *ARD1* is predicted to have a role in transcriptional silencing [24], [25]. These results affirm that our screen was effective for identifying factors that enhance silencing defects of *rtt106*Δ cells.

**Figure 1:**
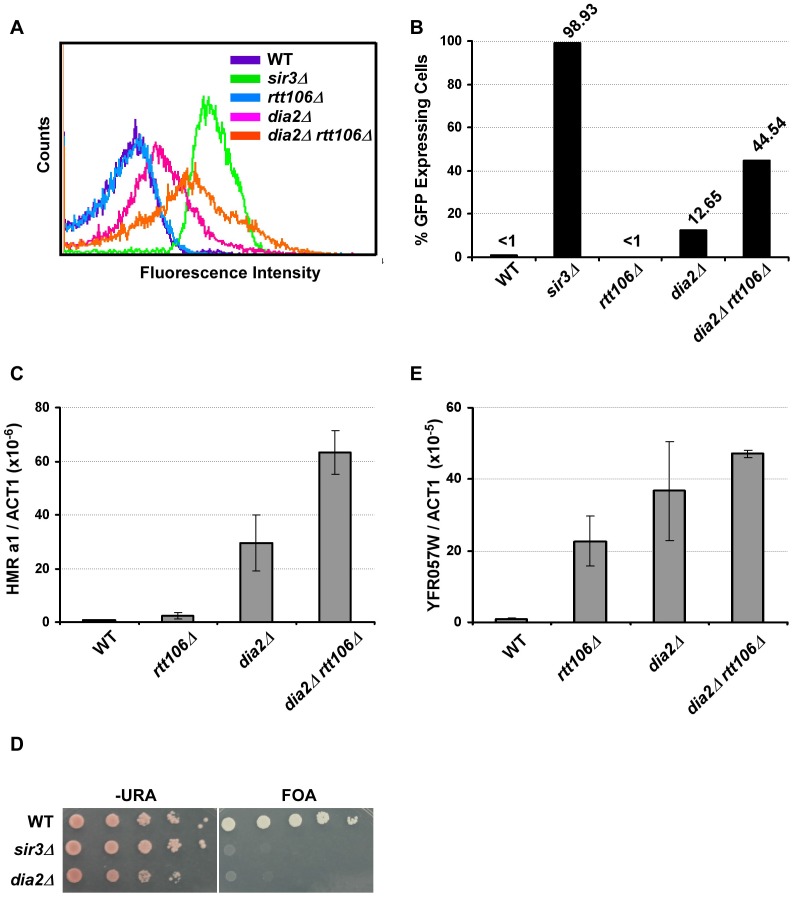
Dia2 functions in parallel with Rtt106 in *HMR* silencing. (A–C) Mutant *dia2*Δ cells have defects in transcriptional silencing at the *HMR* locus. Cells with the indicated genotype were analyzed for GFP expression using flow cytometry (A), and the percentage of cells expressing GFP was reported (B). Wild-type (WT) cells (less than 1%) and cells with *SIR3* deleted (*sir3*Δ, over 95%) were used as standards to set the gate for GFP expression. The flow cytometry profile and percentage of cells expressing GFP are reported from one of three independent experiments. (C) *HMR a1* gene expression is elevated in *dia2*Δ and *dia2Δ rtt106*Δ mutants. RNA was collected from cells of the indicated genotype and reverse transcribed. Expression of the *HMR a1* gene was analyzed via real-time PCR, normalized against the expression of the *ACT1* gene and reported as the average ± standard deviation (s.d.) of two independent experiments. (D–E) Telomere silencing is compromised in *dia2*Δ cells. (D) Telomere silencing was assayed using the *URA3* gene integrated at the left end telomere of chromosome VII and growth on medium containing 5-fluoroorotic acid (FOA). (E) *YFR057W* expression levels were determined by real time RT-PCR and analyzed as described above with the average ± s.d. of two independent experiments shown.

Dia2 is the F-box containing protein of the SCF^Dia2^ ubiquitin E3 ligase involved in DNA replication, and it may also have a role in transcriptional regulation [16], [26]. Therefore, we decided to focus our studies on Dia2. To confirm our results, we deleted *DIA2* from our standard genetic background (W303) and assessed transcriptional silencing at the *HMR* locus using the *HMR::GFP* reporter. Cells defective for transcriptional silencing at the *HMR* locus express GFP and exhibit a rightward shift in the flow cytometry profile as observed for *sir3*Δ cells (Figure 1A). Mutating *DIA2* in *rtt106*Δ cells resulted in a rightward shift compared to single mutant cells (Figure 1A), indicating an increase in the percentage of GFP expressing cells, which was quantified in Figure 1B. Moreover, *dia2*Δ single mutant cells exhibited an elevated percentage of cells expressing GFP compared to wild-type cells, suggesting a role for Dia2 in *HMR* silencing. To validate the flow cytometry analysis, the percentage of cells expressing GFP was also determined using fluorescence microscopy. Among the strains tested, a similar trend was observed using both methods of determining the percentage of cells expressing GFP (Figure S1). To confirm the effect of *dia2*Δ on *HMR* silencing, we determined how the loss of Dia2 affected the expression of the silenced *a1* gene at the *HMR* locus. RNA was collected from single and double mutant cells of the alpha mating type, reverse transcribed, and cDNA analyzed using real-time PCR. Upon normalizing the expression of *a1* to *ACT1, dia2*Δ cells exhibited elevated *a1* gene expression compared to wild-type cells (Figure 1C). Furthermore, *dia2Δ rtt106*Δ had an even greater *a1* gene expression level compared to either the *dia2*Δ or *rtt106*Δ single mutant. These results are consistent with the idea that *DIA2* and *RTT106* function in parallel to regulate transcriptional silencing at the *HMR* locus.

Transcriptional silencing at telomeres and the *HMR* locus utilize similar mechanisms [6]. Moreover, it has been shown that Pof3, the homolog of Dia2 in *S. pombe*, is required for maintaining telomere length and telomeric silencing [27]. We, therefore, determined whether Dia2 in *S. cerevisiae* had a role in telomeric silencing using cells containing the reporter gene, *URA3*, integrated at the left arm of telomere VII. When plated on media containing 5-fluoroorotic acid (FOA) that is toxic to cells that express *URA3*, wild-type cells survive because of telomeric silencing of the *URA3* gene. Cells with telomeric silencing defects (such as *sir3*Δ, used as a control) exhibit growth defects in media containing FOA [24]. We found that *dia2*Δ cells exhibited defects in telomeric silencing compared to wild-type cells (Figure 1D). We confirmed these observed telomeric silencing defects using RT-PCR to assess the expression of *YFR057W*, a gene found at the telomere on the right arm of chromosome VI and known to be silenced via a Sir-mediated mechanism [28]. Compared to wild-type and *rtt106*Δ cells, *dia2*Δ cells exhibited higher *YFR057W* gene expression, suggesting a telomeric silencing defect (Figure 1E). Interestingly, deletion of *RTT106* in *dia2*Δ mutant cells did not significantly increase expression of *YFR057W*, suggesting that deletion of *RTT106* does not exacerbate the telomeric silencing defect of *dia2*Δ cells. These results demonstrate that Dia2 is required for efficient silencing at both the *HMR* locus and telomeres and suggest that Dia2 functions in a pathway parallel to Rtt106 in transcriptional silencing at the *HMR* locus, but not at telomeres.

### The *dia2*Δ mutant exhibits a synthetic interaction with mutations at genes encoding histone chaperones and at histone lysine residues implicated in nucleosome assembly

**Figure 2:**
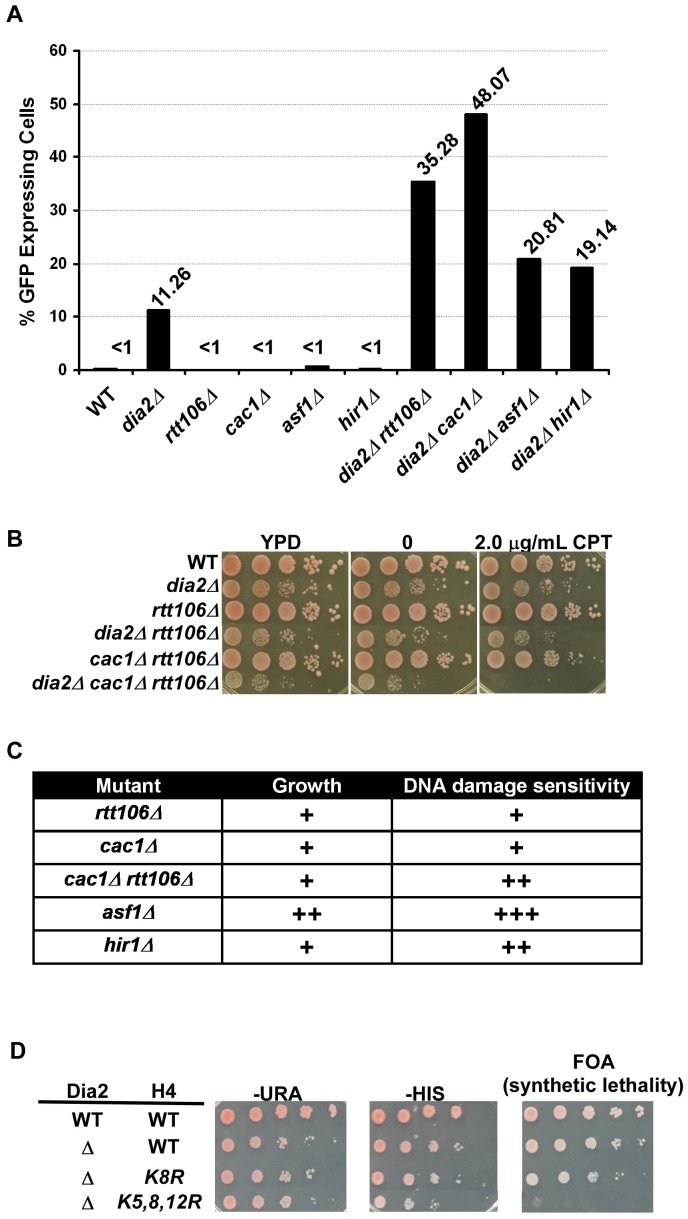
*DIA2* genetically interacts with genes encoding histone chaperones and histone H3–H4 lysine mutants with defects in nucleosome assembly. (A) Dia2 functions in parallel with known H3–H4 histone chaperones in transcriptional silencing at the silent *HMR* locus. Cells of the indicated genotype were assessed for *HMR* silencing as described in Figure 1A–1B. The percentage of cells expressing GFP from one of three independent experiments was reported. (B–C) Dia2 functions in parallel with known H3–H4 histone chaperones in growth and DNA damage sensitivity. (B) Genetic interactions among *DIA2*, *RTT106*, and *CAC1* were assessed by spotting a ten fold series dilution of cells of the indicated genotype onto regular growth media (YPD), media containing DMSO (0) or media with the indicated concentration of camptothecin (CPT). (C) A summary of the genetic analysis of growth and DNA damage sensitivity of histone chaperone mutations combined with deletion of *DIA2*. A ‘+’ indicates a synthetic effect in the double mutant compared to either single mutant alone. Spot assays were performed as described above using different concentrations of CPT and methyl methanesulfonate (MMS). Representative images are shown in Figure S2. (D) The *dia2*Δ mutant is synthetic lethal with mutations at histone H4 lysine residues 5, 8 and 12 (*H4K5,8,12R*). A ten fold serial dilution of cells was spotted onto plates of the indicated media. Both copies of histone genes (*HHT1-HHF1* and *HHT2-HHF2*) were deleted. Wild type H3–H4 was expressed from a *URA3* containing plasmid, whereas the histone H4 mutant (as indicated) was expressed from a plasmid containing the *HIS3* gene. Media containing FOA was used to select for the loss of the *URA3*-containing plasmid and detect synthetic lethality.

In addition to *RTT106*, mutations in *ASF1*, *HIR1* and CAF-1 also result in silencing defects [12], [13]. We, therefore, tested how loss of *DIA2* affected silencing at the *HMR* locus in the absence of each of these H3–H4 histone chaperones. Each double mutant, *dia2Δ asf1*Δ, *dia2Δ cac1*Δ or *dia2Δ hir1*Δ, had a higher percentage of cells expressing GFP compared to the respective single mutants (Figure 2A). These results suggest that Dia2 impacts transcriptional silencing at the *HMR* locus in a pathway parallel to each of the known H3–H4 histone chaperones.

In addition to defects in transcriptional silencing, histone chaperone mutants are sensitive to DNA damaging agents [10], [29], [30]. Moreover, *dia2*Δ cells also exhibit sensitivity to a number of DNA damaging agents [15], [16]. To determine whether the parallel action observed for *DIA2* and H3–H4 histone chaperones extended beyond their function in transcriptional silencing, we tested the growth and DNA damage sensitivity of *dia2*Δ cells containing a mutation at each H3–H4 histone chaperone. The DNA damaging agents camptothecin (CPT) and methyl methanesulfonate (MMS) were used to assess genetic interactions in response to DNA damage. Synthetic interactions in growth and DNA damage sensitivity were observed for *dia2*Δ with each of the histone chaperone mutants tested (Figure 2B, 2C and Figure S2). The most dramatic effects were observed in *dia2Δ asf1*Δ and *dia2Δ cac1Δ rtt106*Δ mutants, with the interaction between *DIA2* and *ASF1* being the most pronounced. Furthermore, *dia2Δ cac1Δ rtt106*Δ cells grew slower on regular growth media (YPD) than *dia2*Δ and *cac1Δ rtt106*Δ cells and showed an increased sensitivity to CPT over *dia2*Δ, *cac1Δ rtt106*Δ and *dia2Δ rtt106*Δ cells. Taken together, these genetic interactions provide support for a role for Dia2 and the SCF^Dia2^ complex in processes linked to nucleosome assembly.

Post-translational modifications on newly synthesized histones work in concert with histone chaperones to regulate nucleosome assembly [9]. For instance, histone H3 lysine 56 acetylation (H3K56Ac), catalyzed by the histone acetyltransferase Rtt109, is important for regulating the interaction between histones and the histone chaperones CAF-1 and Rtt106, and thus, H3K56Ac is a critical regulator of histone deposition [29], [31]. H3 N-terminal tail acetylation, catalyzed by Gcn5 and Rtt109, also serves as an important regulator of nucleosome assembly [32]. Finally, acetylation of histone H4 lysines 5, 8 and 12 (H4K5,8,12Ac), catalyzed by Hat1 and Elp3, has also been implicated in nucleosome assembly [33], [34]. Given the observed genetic interactions between *dia2*Δ and the H3–H4 histone chaperones involved in nucleosome assembly, we, therefore, determined how mutations in these important histone lysine residues affected the growth and CPT sensitivity of *dia2*Δ cells. For each histone mutant, the acetylated lysine residues were mutated to arginine to mimic the unacetylated state. We observed that *dia2*Δ cells showed significant growth defects and increased sensitivity towards CPT when combined with *H3K56R*, *H4K5,12R* and *H3K9,14,18,23,27R* (Table 1). Notably, we were unable to construct the *dia2Δ H4K5,8,12R* mutant via plasmid shuffling, suggesting that *dia2*Δ exhibited a synthetic lethal interaction with mutations at H4 lysines 5, 8 and 12 (Table 1 and Figure 2D). To confirm this result, wild-type and *dia2*Δ cells expressing wild-type histones H3–H4 from a uracil (URA3) containing plasmid were transformed with either wild-type or mutant forms of H3-H4K5, 8, 12 on a plasmid with a histidine (HIS) selection marker. The wild-type H3–H4 histone plasmid (URA) could not be lost in *dia2Δ H4K5,8,12R* cells (as no growth was observed when these cells were plated on FOA medium), whereas this plasmid was readily lost in *dia2*Δ or *dia2Δ H4K8R* cells (Figure 2D). This suggests that Dia2 functions in parallel with acetylation of H4 lysine residues 5, 8 and12 in maintaining cell viability. Together, these genetic analyses provide further evidence supporting the idea that the SCF^Dia2^ E3 ligase has a role in a process linked to nucleosome assembly.

**Table 1:** Effect of histone lysine mutations on the growth and DNA damage sensitivity of *dia2*Δ cells.

Mutant	Growth defect with *dia2*Δ	DNA damage sensitivity with *dia2*Δ
*H3K56R*	++	++
*H4K5,12R*	+	++
*H4K5,8,12R*	synthetic lethal	n/a
*H4K8R*	+	++
*H4K16R*	−	−
*H4K8,16R*	+	++
*H4K91R*	−	−
*H3K9R*	−	+
*H3K14R*	−	+
*H3K9,14R*	−	+
*H3K9,14,18,23,27R*	+	+

The *dia2*Δ mutant, each histone mutant and their corresponding double mutant, containing both the *dia2*Δ mutation and the indicated histone mutation, were assayed for cell growth and sensitivity towards the DNA damaging agent camptothecin (CPT). A ‘−’ represents no effect on growth or DNA damage sensitivity over the individual single mutants. A ‘+’ represents a synthetic effect in growth on regular media (YPD) or DNA damage sensitivity (growth on medium containing CPT). More+s indicate a more dramatic synthetic effect in comparison with other strains tested. Note that the *dia2Δ H4K5,8,12R* mutant is synthetic lethal, and therefore, DNA damage sensitivity could not be assessed (n/a).

### The F-box and LRR domain of Dia2 are required for transcriptional silencing

**Figure 3:**
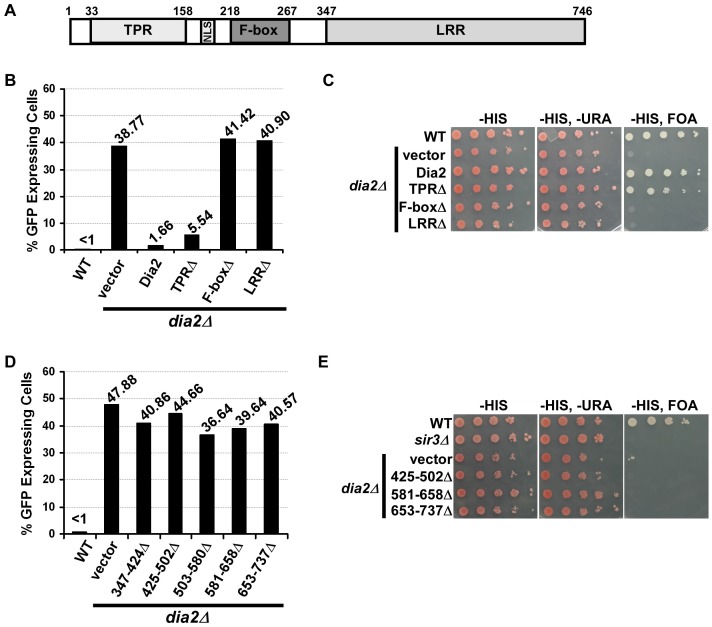
Dia2's F-box and LRR domains are indispensable for transcriptional silencing. (A) A schematic drawing of the domain structure of Dia2. Dia2 contains a tetratricopeptide repeat (TPR) region, nuclear localization signal (NLS), F-box and leucine rich repeat (LRR) domain. (B) The Dia2 F-box and LRR domains are important for silencing at the *HMR* locus. The *dia2*Δ mutant cells containing the *HMR::GFP* reporter were transformed with empty vector or a plasmid expressing WT or different *dia2* mutants, and the percentage of cells expressing GFP was determined as in Figure 1B. Note that the percentage of cells expressing GFP in *dia2*Δ cells was higher when using selective media compared to cells growing in YPD. (C) The Dia2 F-box and LRR domains are important for telomere silencing. The *dia2*Δ mutants containing the *VIIL-URA3* reporter were transformed with empty vector or a plasmid expressing the indicated *dia2* mutant and spotted onto selective media (-HIS) and media containing FOA (-HIS, FOA) to assay silencing. (D–E) The Dia2 LRR domain is important for *HMR* silencing (D) and telomere silencing (E). Assays were performed as described in B and C.

Dia2 contains three primary functional domains: an N-terminal tetratricopeptide repeat (TPR) region, an F-box domain and a C-terminal leucine rich repeat (LRR) domain (Figure 3A). The TPR region is important for Dia2's localization to the replisome [14], [17]. The F-box domain is required for Dia2 ubiquitylation activity, as the F-box, in general, facilitates interactions with other SCF^Dia2^ components [15], [16]. The LRR region is proposed to be an interaction motif for substrates of the SCF^Dia2^ complex [17]. To gain further insight into Dia2's role in transcriptional silencing, we deleted each of the three domains of Dia2, expressed these *dia2* mutants in *dia2*Δ cells and analyzed transcriptional silencing at the silent *HMR* locus and the telomere. Expression of Dia2 in *dia2*Δ cells restored the silencing at the *HMR* locus, as the percentage of cells expressing GFP was similar to wild-type cells (Figure 3B). Expression of the Dia2-TPRΔ domain mutant also restored *HMR* silencing. Interestingly, deletion of the Dia2 F-box (F-boxΔ) or LRR domain (LRRΔ) did not rescue the *HMR* silencing defect. These results suggest that the Dia2 F-box and LRR domains are indispensable for silencing at the *HMR* locus.

Similar to the effects observed at the *HMR* locus, the expression of full-length Dia2 in *dia2*Δ cells containing the *URA3* reporter at telomere VIIL rescued the telomere silencing defects of *dia2*Δ cells to that of wild-type cells, and expression of Dia2 lacking the TPR region (TPRΔ) resulted in an almost complete rescue of *dia2*Δ telomere silencing defects (Figure 3C). In contrast, expression of Dia2 lacking the F-box (F-boxΔ) or the LRR region (LRRΔ) was unable to rescue the telomere silencing defects of *dia2*Δ cells.

While the expression of the TPRΔ and F-boxΔ mutants was similar to that of full length Dia2 in *dia2*Δ cells (Figure S3), expression of the Dia2 LRRΔ mutant was much less than the other mutants (data not shown), most likely due to instability of the shortened form of the protein. Therefore, we made five additional Dia2 mutants, each with deletion of approximately 75 amino acids of the LRR domain: LRR (amino acids 347–424)Δ, 425–502Δ, 503–580Δ, 581–658Δ, and 653–737Δ. Expression of these five mutants was, for the most part, similar to full length Dia2 and other Dia2 mutant forms (Figure S3). Importantly, *dia2*Δ cells expressing each of the LRR mutants exhibited defects in *HMR* (Figure 3D) and telomere silencing (Figure 3E) similar to *dia2*Δ cells transformed with empty vector. Together, these results suggest that silencing defects displayed in cells expressing *dia2* mutants lacking the LRR or F-box are unlikely due to reduced expression of these mutant proteins. Instead, the F-box domain, essential for Dia2's role in protein ubiquitylation, and LRR region, predicted to be important for substrate recognition, are indispensable for SCF^Dia2^'s role in transcriptional silencing, suggesting that SCF^Dia2^'s role in silencing is likely mediated through its ability to ubiquitylate a substrate involved in transcriptional silencing.

### Sir3 and Sir4 are mis-localized in *dia2*Δ cells

**Figure 4:**
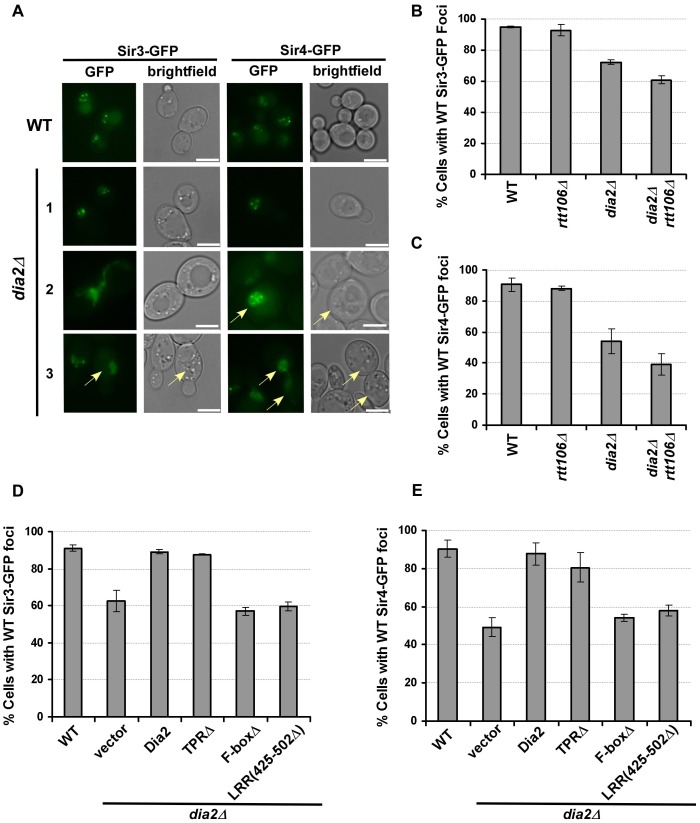
Sir3 and Sir4 are mislocalized in *dia2*Δ cells and cells expressing *dia2* mutants lacking the Dia2 F-box or LRR domain. (A–C) Sir3 and Sir4 are mislocalized in *dia2*Δ and *dia2Δ rtt106*Δ cells. (A) Representative images of Sir3-GFP or Sir4-GFP foci in WT and *dia2*Δ cells. Cells expressing Sir3-GFP and GFP-Sir4 exhibited nuclear foci in WT cells, and this pattern is lost in some *dia2*Δ cells. Note that *dia2*Δ cells are larger in size than wild-type cells (images were taken under the same magnification). The scale bar represents 5 µm. Cells that exhibited Sir protein localization defects as described in the text were marked by arrows. (B–C) Quantification of the percentage of *dia2*Δ cells that had WT-like Sir3-GFP foci (B) and WT-like Sir4-GFP foci (C). (D–E) Cells expressing *dia2* mutants lacking the Dia2 F-box and LRR domains exhibit defects in the localization of Sir3 (D) and Sir4 (E). For each experiment, at least 100 cells were counted for each genotype, and the average ± s.d. percentage of cells expressing WT-like foci from at least two independent experiments was reported.

Sir proteins serve as structural components of yeast silent chromatin [6]. Sir3 and Sir4 form four to five foci at the nuclear periphery, which reflects the clustering of yeast telomeres [35]–[37]. Thus, we tested whether loss of Dia2 affected the localization of Sir3 and Sir4, and therefore, silent chromatin structure, using fluorescence microscopy. As reported, wild-type cells expressing Sir3-GFP or GFP-Sir4 formed foci at the nuclear periphery [11], [37]. While some *dia2*Δ cells had Sir3-GFP or Sir4-GFP foci patterns similar to wild-type cells (Figure 4A, *dia2*Δ, panel 1), a considerable percentage of *dia2*Δ mutant cells lost proper localization of Sir3 (Figure 4B) and Sir4 (Figure 4C) in *dia2*Δ or *dia2Δ rtt106*Δ cells. In the case of Sir3-GFP foci, some mutant cells had large areas of fluorescence without distinct foci (Figure 4A, *dia2*Δ panels 2 and 3). A commonly observed Sir4 pattern was multiple small foci (>8) that were relatively non-distinct (Figure 4A, *dia2*Δ panel 2), in addition to cells containing areas of fluorescence with no distinct foci (Figure 4A, *dia2*Δ, panel 3). Because the *dia2*Δ mutation did not affect the protein levels of Sir3 and Sir4 to a significant degree (Figure S4), the mis-localization of Sir3 and Sir4 observed in *dia2*Δ mutant cells is likely due to changes in telomeric chromatin structure.

To determine whether the localization of Sir3-GFP and Sir4-GFP was dependent upon particular Dia2 domains, we expressed the Dia2 mutants lacking specific domains (see Figure 3) in *dia2*Δ cells. Expression of full-length Dia2 or Dia2 TPRΔ restored the percentage of cells containing wild-type Sir3-GFP or Sir4-GFP foci closer to the percentage observed for wild-type cells, whereas cells expressing *dia2* mutants lacking the F-box (F-boxΔ) or a portion of the LRR domain [LRR(425–502Δ) and LRR(581–658Δ)] did not (Figure 4D–4E and data not shown). Thus, the F-box and LRR regions are important for proper localization of Sir3 and Sir4, consistent with the idea that Dia2's role in transcriptional silencing is dependent upon its ubiquitylation activity.

### Sir4 binding at the *HMR a1* gene locus is elevated in *dia2Δ rtt106*Δ cells

**Figure 5:**
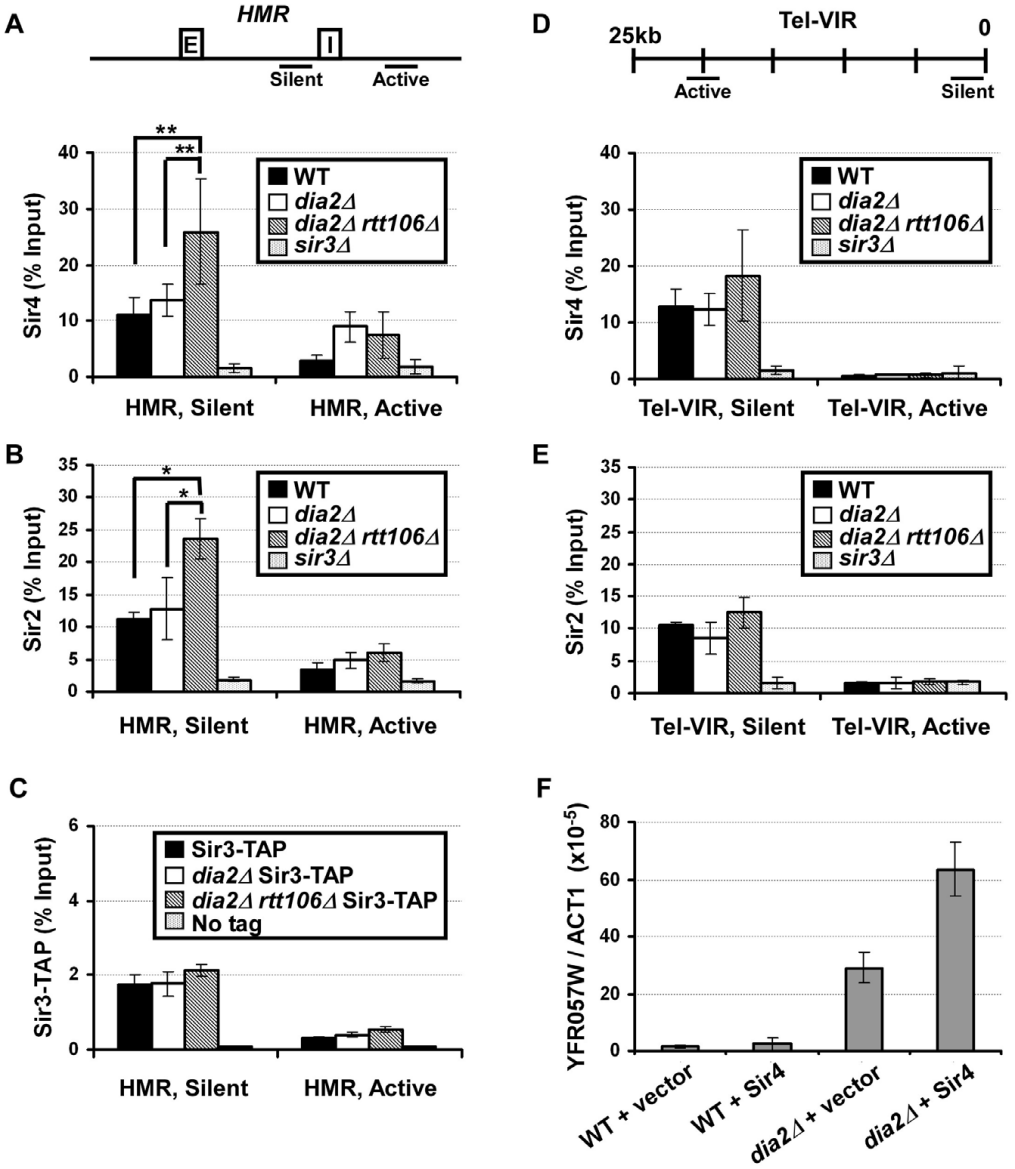
Sir protein binding at the *HMR* locus is altered in *dia2Δ rtt106* Δ** cells.** (A) Sir4 binding at the *HMR* silent chromatin locus is increased in *dia2Δ rtt106*Δ cells. ChIP assays were performed using an antibody against Sir4, and the resulting DNA was quantified via real time PCR using primers amplifying the *a1* gene located at the *HMR* silent mating type cassette (HMR, silent,) and a region outside of the silent chromatin boundary (HMR, active). The upper panel indicates the location of primers at the *HMR* locus, and E and I represent the E and I silencers at the *HMR* locus. (B) Sir2 binding is increased at *HMR* silent chromatin in *dia2Δ rtt106*Δ cells. ChIP assays were performed as described in (A) using antibodies against Sir2. (C) Sir3 binding at the *HMR* locus is not altered in *dia2*Δ mutants. Sir3-TAP ChIP was performed using IgG sepharose. (D) Sir4 binding to telomeric silent chromatin is not significantly altered in *dia2Δ rtt106*Δ cells. ChIP assays were performed as described in (A), and the ChIP DNA was analyzed using PCR primers amplifying a fragment 0.77 kb from the telomere end of the right arm of chromosome VI right (Tel-VIR, silent) or 20 kb from the telomere end (Tel-VIR, active). The PCR primer locations are indicated in the upper panel. (E) Sir2 binding to telomeric heterochromatin is not significantly altered. ChIP samples from (B) were analyzed for the telomere positions. ChIP data is presented as the average ChIP signal (as the percentage of input) ± s.d. from at least three independent experiments. * represents a p-value <0.05, ** indicates a p-value <0.01 obtained using the student's t-test. (F) Ectopic expression of Sir4 further compromises telomeric silencing in *dia2*Δ cells. RNA was extracted from WT and *dia2*Δ cells transformed with empty vector or a centromere plasmid for expression of Sir4, and the expression of *YFR057W* was analyzed as described in Figure 1E.

To further analyze how the *dia2*Δ mutation affects the binding of Sir proteins to chromatin, chromatin immunoprecipitation (ChIP) assays were performed in unsynchronized wild-type, *dia2Δ, dia2Δ rtt106*Δ and control cells (*sir3*Δ) using antibodies against Sir4, Sir2, and Sir3. ChIP DNA was analyzed using real-time PCR with primers amplifying different positions at the *HMR* locus or the telomere region on the right arm of chromosome VI (Tel-VIR). Consistent with published results, Sir protein binding at silent chromatin loci was lost in *sir3*Δ cells, and more Sir proteins bound to silent chromatin than the corresponding active chromatin loci tested at both the *HMR* locus and telomere (Figure 5 and Figure S5) [11]. Compared to wild-type cells, *dia2*Δ cells exhibited a slight elevation in Sir4 binding at *HMR* silent chromatin (*a1* gene, HMR silent) (Figure 5A), whereas *dia2Δ rtt106*Δ cells had a much larger and significant increase in Sir4 binding at the silent *HMR* locus than wild-type and *dia2*Δ cells (Figure 5A). Similarly, we also observed significantly more Sir2 binding at the *HMR* locus in *dia2Δ rtt106*Δ mutant cells compared to wild-type and *dia2*Δ mutant cells (Figure 5B). In contrast, Sir3 binding to the silent *HMR* locus was not altered to a significant degree in either *dia2*Δ or *dia2Δ rtt106*Δ cells when chIP was performed using an antibody against endogenous Sir3 (Figure S5A) or performed using IgG beads in strains in which Sir3 was tagged with a tandem affinity purification (TAP) tag (Sir3-TAP)(Figure 5C). Notably, the *dia2*Δ or *dia2Δ rtt106*Δ mutation did not affect mRNA levels of Sir4 or Sir2 (Figure S6), suggesting that the increase in Sir4 and Sir2 proteins at the *HMR* locus is not likely due to increased gene transcription in mutant cells. In addition, the overall protein levels of Sir3 and Sir4 were not altered to a detectable degree in cell extracts (Figure S4). Therefore, the observed elevation in Sir4 and Sir2 at the silent *HMR* locus in *dia2*Δ cells is not likely due to increased steady-state levels of Sir proteins and is likely due to elevated Sir2 and Sir4 levels on chromatin.

Sir protein binding was also assessed at telomeric silent chromatin. No significant change in Sir4 binding was detected at telomeric silent chromatin (Tel-VIR, silent) in *dia2*Δ cells compared to wild-type cells (Figure 5D). Sir4 binding to the telomeric silent chromatin in *dia2Δ rtt106*Δ cells was not increased significantly compared to wild-type (p-value for three independent experiments is 0.1). Thus, levels of Sir4 proteins are unlikely altered at telomeres in *dia2Δ rtt106*Δ mutant cells compared to wild-type cells. These results are consistent with the result in Figure 1 showing that deletion of *RTT106* synergistically increases the silencing defect of *dia2*Δ cells at the HMR locus, but not the telomere. Sir2 (Figure 5E) and Sir3 (Figure S5B and S5C) binding to telomeric silent chromatin was not altered to a detectable degree in either *dia2*Δ or *dia2Δ rtt106*Δ cells compared to wild-type cells. Taken together, it is likely that mutations in Dia2 alter the chromatin binding of Sir4 and Sir2, but not Sir3, in *dia2Δ rtt106*Δ cells, and these imbalanced alterations in Sir protein binding to silent chromatin may contribute to the silencing defects observed for *dia2*Δ and *dia2Δ rtt106*Δ cells, especially at the silent *HMR* locus.

### Expression of Sir4 in *dia2*Δ mutant cells leads to synergistic loss of telomeric silencing

Overexpression of Sir4 is known to result in silencing defects, most likely due to disruptions in Sir protein stoichiometry [38], [39]. To further test whether the effect of *dia2*Δ on silencing is due to altered Sir4 protein levels on chromatin, we investigated the effects of exogenous expression of Sir4 on telomeric silencing, as transcriptional silencing at telomeres is perturbed more easily than that at the *HMR* locus [40]. Wild-type and *dia2*Δ cells were transformed with a centromere plasmid for expression of Sir4 under the control of its own promoter. RNA was then extracted for quantitative RT-PCR analysis of *YFR057W* expression. While ectopic expression of Sir4 in wild-type cells did not result in obvious changes in expression of *YFR057W*, Sir4 expression in *dia2*Δ cells resulted in over a two-fold increase in *YFR057W* expression compared to *dia2*Δ cells transformed with empty vector (Figure 5F). This suggests that telomeric silencing in *dia2*Δ cells is more sensitive to changes in Sir4 levels than that of wild-type cells. These results are consistent with the idea that altered Sir4 levels at chromatin contribute to the silencing defects observed in *dia2*Δ cells.

### SCF^Dia2^ ubiquitylates Sir4

**Figure 6:**
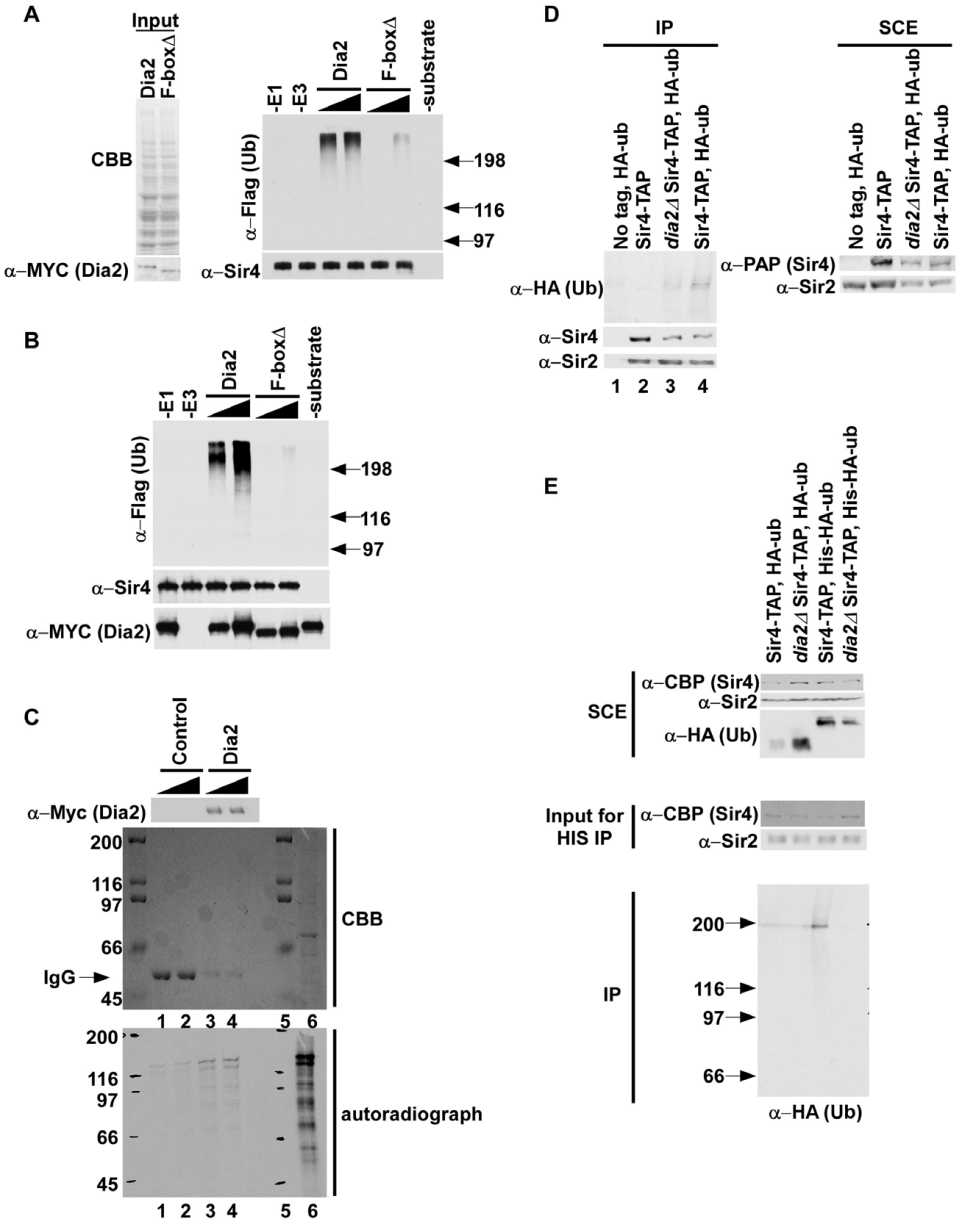
SCF^Dia2^ ubiquitylates Sir4 *in vitro* and *in vivo*. (A–B) Dia2 ubiquitylates Sir4 *in vitro* (A) (left panel) Cell extracts prepared from full length MYC-Dia2 or MYC-Dia2 F-boxΔ (F-boxΔ) cells were normalized using Coomassie staining (CBB, upper panel) and Western blot analysis using antibodies against the MYC epitope (bottom panel). (right panel) Two different amounts of extract were used for *in vitro* ubiquitylation assays using Sir4-TAP purified from *dia2*Δ cells as substrate, Flag-ubiquitin (Flag-ub), E1, and E2 (Cdc34). Following the ubiquitylation reaction, Sir4-CBP proteins were purified using calmodulin beads, and ubiquitylated species were detected via Western blot using Flag antibody. Sir4 were detected by Western blot using antibody against Sir4. (B) Ubiquitylation reactions were carried out as described in A except purified MYC-Dia2 or MYC-Dia2 F-boxΔ was used as E3. (C) Sir4 binds the SCF^Dia2^ complex *in vitro*. Binding studies were performed using control beads, prepared by incubating beads with antibodies against the Myc epitope, or beads containing the MYC-Dia2 complex prepared as in B and two different amounts of in vitro translated ^35^S-methione labeled Sir4 (lane 6, ^35^S-Sir4 input). The presence of the SCF^Dia2^ in binding samples was detected by Western blot (upper panel) using antibodies against the Myc epitope. In addition, binding samples were resolved by SDS-PAGE, stained with Coomassie (CBB, middle panel) and bound ^35^S-Sir4 was detected via autoradiography (lower panel); Lane 5 is a protein marker. (D–E) Sir4 is ubiquitylated *in vivo*, and this ubiquitylation is decreased in *dia2*Δ cells. (D) Sir4-TAP was purified from cells of the indicated genotype either with or without expression of HA-ubiquitin (HA-ub) using tandem affinity purification. Ubiquitylated species were detected using antibodies against the HA epitope. Right panel: SCE, soluble cell extract. (E). Sir4-TAP was purified from wild-type or *dia2*Δ mutant cells expressing either HA-ubiquitin or His-HA-Ubiquitin using IgG sepharose. After cleavage with TEV protease, the eluted proteins were denatured and purified using Ni-NTA beads. Purified ubiquitylated species were detected by Western blot using antibodies against HA.

Since *dia2Δ rtt106*Δ cells exhibited elevated Sir4 binding at silent chromatin, we hypothesized that SCF^Dia2^ may ubiquitylate Sir4. To test this idea, we first determined whether cell extracts prepared from cells expressing MYC-Dia2 or MYC-Dia2 F-boxΔ integrated at the endogenous *DIA2* locus (Figure 6A, left panel) could ubiquitylate Sir4 purified from *dia2*Δ cells using TAP purification. Briefly, Flag–ubiquitin (Flag-Ub), E1, E2 (Cdc34) and the respective whole cell extract (E3) were incubated with Sir4-CBP (calmodulin binding peptide, part of the TAP tag). As negative controls, reaction mixtures containing all components except E1, E3 (cell extract) or substrate were assembled. Following ubiquitylation, Sir4 was pulled down using calmodulin beads, and ubiquitylated species were detected via Western blot using antibodies against the Flag epitope. More Sir4-associated ubiquitylated species were detected in the reactions using the extracts from full length Dia2 than from those using extracts from cells expressing Dia2 lacking the F-box domain (Figure 6A, right panel). No ubiquitylated species were detected in control reactions lacking E1, E3 or Sir4 (substrate). Thus, SCF^Dia2^ ubiquitylates Sir4 and/or a Sir4-associated protein *in vitro*.

To test this idea further, the SCF^Dia2^ complex was purified from yeast cells expressing MYC-Dia2 and MYC-Dia2 F-boxΔ and used to ubiquitylate Sir4 purified from yeast cells as described in Figure 6A. More ubiquitylated species were detected in reactions containing purified SCF^Dia2^ (Dia2) than those containing the complex purified using the Dia2 F-box mutant (F-boxΔ) (Figure 6B). Taken together, these experiments indicate that SCF^Dia2^ ubiquitylates Sir4, or a protein associated with Sir4, *in vitro*, and this ubiquitylation depends on the F-box domain of Dia2.

To provide additional evidence that SCF^Dia2^ ubiquitylates Sir4, we tested whether Sir4 bound to SCF^Dia2^ *in vitro*. Beads containing the MYC-Dia2 complex purified for *in vitro* ubiquitylation reactions in Figure 6B or control beads were incubated with two different amounts of *in vitro* translated ^35^S-methionine labeled Sir4. After the beads were washed, bound ^35^S-Sir4 was detected via autoradiography, and the presence of the MYC-Dia2 was detected via Western blot using antibodies against the Myc epitope. Compared to control reactions, more ^35^S-Sir4 signal was detected in samples containing the MYC-Dia2 complex (Figure 6C, compare lanes 3–4 to lanes 1–2 of lower panel) despite the fact that more MYC antibodies (IgG) could be detected by CBB staining in control samples (Figure 5C, middle panel) than samples containing Myc-Dia2 (Figure 5C, upper panel) These data further support the idea that Sir4 is a substrate of the SCF^Dia2^ complex.

Next, we asked whether Sir4 is ubiquitylated *in vivo* and whether this ubiquitylation depends on Dia2 using our published procedures [41]. Briefly, Sir4-TAP was purified from wild type or *dia2*Δ cells transformed with a plasmid expressing HA-tagged ubiquitin (HA-Ub). Following TAP purification, ubiquitylated species were detected by Western blot with antibodies against the HA epitope. In wild-type (Sir4-TAP) cells, ubiquitylated protein species co-purified with Sir4, and these immunoprecipitated species were specific as no HA signal could be detected in controls (a strain without Sir4-TAP but containing HA-Ub or a strain with Sir4-TAP but no HA-Ub)(Figure 6D, lanes 1 and 2, respectively). Importantly, these ubiquitylated species were notably reduced in *dia2*Δ cells compared to wild-type cells, despite equal levels of purified Sir4 (Figure 6D, compare lane 4 to lane 3). These results suggest that the SCF^Dia2^ complex ubiquitylates Sir4 *in vivo*. Interestingly, we observed similar amounts of Sir2 co-purified with Sir4 in wild-type cells and *dia2*Δ cells, suggesting that the Sir2-Sir4 interaction is not affected to a detectable degree in *dia2*Δ mutant cells (Figure S7).

To further confirm that Sir4 is ubiquitylated *in vivo*, we purified ubiquitylated species using a two-step purification procedure. First, Sir4-TAP was purified from wild-type and *dia2*Δ mutant cells expressing either HA-tagged ubiquitin or His-HA tagged ubiquitin (Figure 6E). The associated ubiquitylated species were then purified under denaturing conditions using Ni-NTA beads that bound to His-ubiquitin. A band with a similar size to Sir4 was detected from wild-type cells expressing His-HA ubiquitin, but not from *dia2*Δ cells expressing His-HA tagged ubiquitin. In addition, little signal was detected from cells expressing HA-ubiquitin (lanes 1 and 2), suggesting that co-purification of Sir4 with ubiquitin under denatured conditions was specific (Figure 6E). Together, these data strongly support the conclusion that Sir4 is a substrate for SCF^Dia2^.

### Sir4 binding and transcriptional silencing at telomeres is altered during the cell cycle in a Dia2-dependent manner

**Figure 7:**
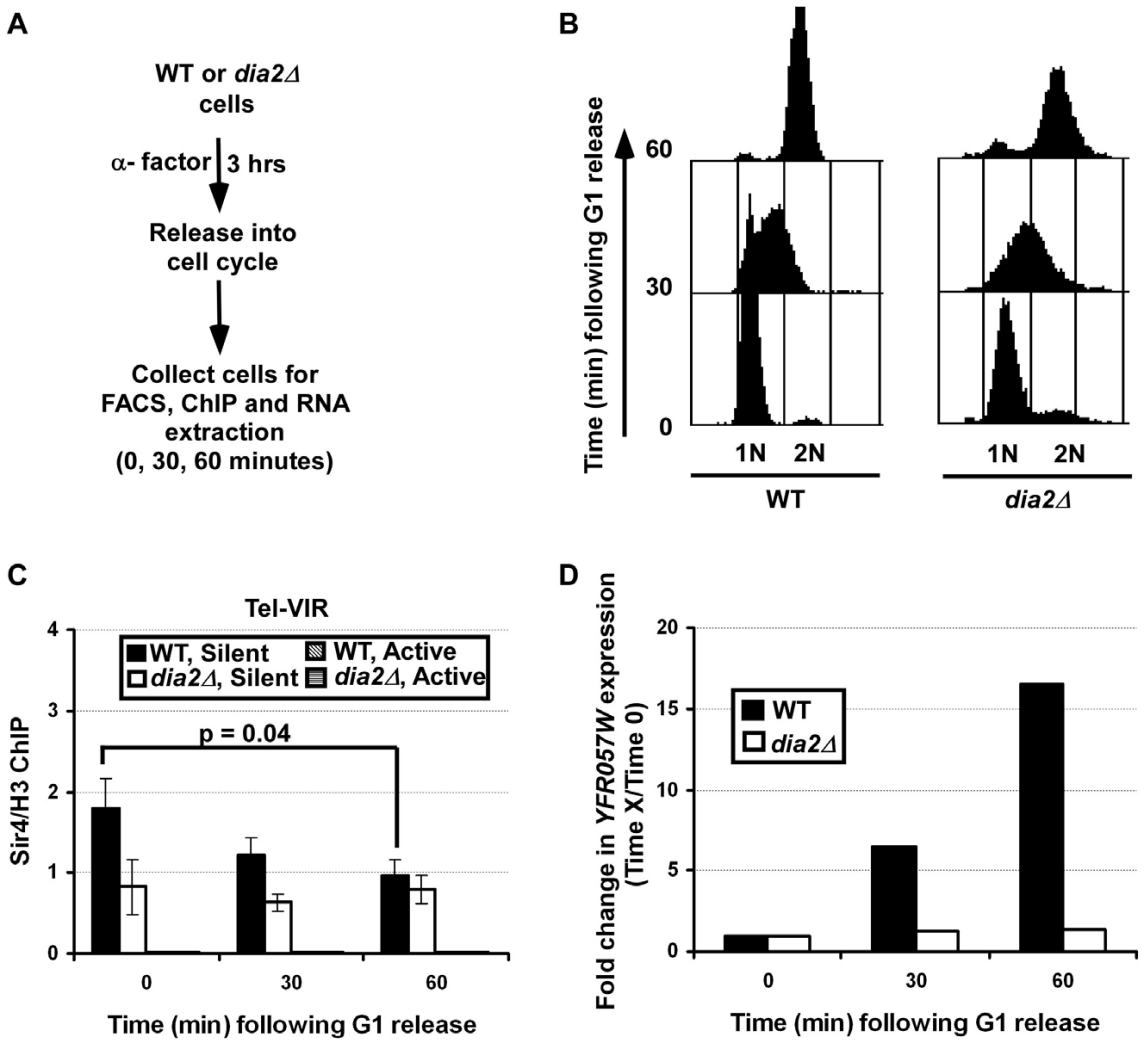
Sir4 binding at telomeric silent chromatin and expression of a telomeric gene are regulated during the cell cycle in a Dia2-dependent manner. (A) Schematic representation of the experiments to test Sir4 binding and gene expression at telomeric silent chromatin during the cell cycle. Briefly, WT or *dia2*Δ cells were arrested at G1 with α-factor. Samples were collected for analysis of DNA content (B), Sir4 ChIP (C) and (D) gene expression at 0, 30 and 60 minutes following released into cell cycle. (B) Cell cycle analysis of WT and *dia2*Δ cells collected in the experiment described in A. DNA was stained using propidium iodide and analyzed using flow cytometry. (C) Sir4 binding is reduced at telomere silent chromatin as cells progress through the cell cycle, whereas Sir4 levels in *dia2*Δ mutant cells are not changed during the cell cycle. At each time point, cells were collected for ChIP assay using antibodies against Sir4 and histone H3. ChIP DNA was analyzed by real time PCR using PCR primers amplifying both silent and active chromatin loci as described in Figure 5D. The Sir4 ChIP signal was normalized against that of H3. The data presented is the average ± s.d. of three independent experiments with the p-value indicated as determined using the student's t-test. (D) The expression of the *YFR057W* increases as the cell cycle progresses. RNA was isolated from cells collected at each time point and reverse transcribed. The expression of *YFR057W* was analyzed using quantitative real-time PCR and was normalized against the expression of *ACT1* as described in Figure 1E. Data is presented as the ratio of the relative expression of *YFR057W* at each indicated time point to the *YFR057W* expression at the G1 phase (0) time point for the respective cell type (thus, *YFR057W* expression at time 0 is 1 for both genotypes). The average calculated from two independent experiments is shown.

It has been shown that heterochromatin is dynamically regulated during S phase of the cell cycle in mammalian cells. During mitosis, phosphorylation of histone H3 serine 10 by Aurora B kinase displaces HP1 from heterochromatin [42], [43]. In *S. pombe*, this dynamic loss of HP1 protein from heterochromatin is proposed to facilitate transcription of siRNA required for the re-establishment of heterochromatin during S phase [44]. Therefore, we asked whether Sir4 proteins at chromatin are also dynamically regulated during mitotic cell division and whether this regulation depends on Dia2. Briefly, wild-type and *dia2*Δ mutant cells were arrested at G1 using α-factor and then released into the cell cycle. Cells were collected at 0, 30 and 60 minutes following release for analysis of DNA content by flow cytometry (Figure 7B), for ChIP assays using Sir4 antibodies (Figure 7C) and for analysis of telomere gene expression by RT-PCR (Figure 7D). Flow cytometry analysis indicated that both wild-type and *dia2*Δ mutant cells collected at 0, 30 and 60 minutes were predominantly at G1, S and G2/M phase of the cell cycle, respectively (Figure 7B). In wild-type cells, Sir4 binding at telomeric silent chromatin was significantly reduced as the cells proceeded from G1 to S and G2/M phases (Figure 7C). In contrast, Sir4 binding was not altered at telomeric silent chromatin in *dia2*Δ cells as the cells progressed through the cell cycle.

To determine whether the observed changes in Sir4 binding affect silencing at telomeres, RT-PCR was performed to assess the expression of the telomere gene, *YFR057W*, as the cells progressed through the cell cycle. Consistent with Figure 1E, *dia2*Δ cells exhibited higher expression of *YFR057W* at all time points compared to wild-type cells (data not shown). When normalized against the expression of *YFR057W* at G1, we observed that there was an increase in gene expression of the telomere gene, *YFR057W* as cells progressed from G1 through to G2/M phase in wild-type cells (Figure 7D). Interestingly, only minor fluctuations in *YFR057W* expression were observed in *dia2*Δ cells. Together, these results suggest that Sir4 proteins at silent chromatin in budding yeast are regulated during the cell cycle, and this regulation is dependent on Dia2. Given our observation of SCF^Dia2^'s role in Sir4 ubiquitylation, we suggest that Sir4 ubiquitylation, mediated by SCF^Dia2^, regulates the binding of Sir4 at silent chromatin during the cell cycle.

## Discussion

In a yeast genetic screen for genes that function in parallel with *RTT106* in transcriptional silencing, we found that deletion of *DIA2*, which encodes the F-box protein of the SCF^Dia2^ E3 ubiquitin ligase complex with a role in DNA replication, enhances silencing defects of *rtt106*Δ mutant cells. Epistasis analysis suggests that *DIA2* functions in parallel with CAF-1, Hir1 and Asf1, three other H3–H4 histone chaperones, in transcriptional silencing at the *HMR* locus, growth and the response to DNA damaging agents. In addition, localization of Sir proteins to silent chromatin loci is altered in *dia2*Δ mutant cells, and this alteration depends on Dia2's role in protein ubiquitylation. Furthermore, we show that SCF^Dia2^ ubiquitylates Sir4 *in vitro* and *in vivo* and that Sir4 levels at silent chromatin are dynamically regulated during the cell cycle. Remarkably, this dynamic regulation is compromised in *dia2*Δ mutant cells. These results reveal a novel role for SCF^Dia2^ in transcriptional silencing and suggest that SCF^Dia2^ functions in silencing in part by ubiquitylating Sir4, which could serve as a mechanism to regulate Sir4 chromatin binding during the cell cycle.

### A role for SCF^Dia2^ in nucleosome assembly

The SCF^Dia2^ complex is known to have a role in DNA replication [16]. However, how SCF^Dia2^ functions in DNA replication is not clear. Our genetic analysis suggests that SCF^Dia2^ may function in DNA replication-coupled nucleosome assembly. First, we show that the *dia2*Δ mutation exhibits synthetic defects in growth and sensitivity towards DNA damaging agents when combined with mutations in CAF-1, Asf1 and Rtt106, histone H3–H4 chaperones known to be involved in replication-coupled nucleosome assembly. Second, we show that the *dia2*Δ mutation exhibits synthetic defects with mutations at lysine residues of histones H3 and H4 known to be involved in the regulation of replication-coupled nucleosome assembly. Surprisingly, the *dia2*Δ mutation is synthetic lethal with mutations at H4 lysine residues 5, 8 and 12. Acetylation of these lysine residues occurs on newly synthesized H4 and is conserved from yeast to human cells [9]. Interestingly, *DIA2* shares many genetic interactions with *RTT101* [45], another ubiquitin ligase functioning in DNA replication [41], [46]. Genetic analysis using the epistatic miniarray profile (E-MAP) approach indicates that Rtt101 functions in the same genetic pathway as Rtt109, the histone H3 lysine 56 acetyltransferase known to be involved in DNA replication-coupled nucleosome assembly [29], [31], [47]. These results, combined with ours presented here, suggest that the SCF^Dia2^ ubiquitin E3 ligase may function in nucleosome assembly. Further investigation is needed to determine Dia2's exact role in this process.

### A role for SCF^Dia2^ in transcriptional silencing

Using RT-PCR and reporter genes integrated at the *HMR* locus and telomeres, we show that Dia2 is needed for efficient transcriptional silencing at both the *HMR* locus and telomeres. Cells with defects in nucleosome assembly are known to affect transcriptional silencing [11], [12], [48]. Because genetic studies presented here suggest that Dia2 has a role in nucleosome assembly, it is possible that Dia2 impacts silencing through its role in nucleosome assembly. However, we have presented several lines of evidence supporting the idea that SCF^Dia2^ impacts transcriptional silencing, at least partly through ubiquitylation of Sir4. First, we show that the role of SCF^Dia2^ in transcriptional silencing depends on the Dia2 functional domains (the F-box and LRR) involved in protein ubiquitylation. Second, SCF^Dia2^ ubiquitylates Sir4 *in vitro* and *in vivo* and interacts with Sir4 *in vitro*. Third, in wild-type cells, the levels of Sir4 at the silent telomere locus are reduced when cells proceed from G1 to S and G2/M phase of the cell cycle, and this regulation is not observed in *dia2*Δ mutant cells. Importantly, the reduction of Sir4 at telomere silent chromatin correlates with increased transcription of *YFR057W*, a gene located near a telomere, during the cell cycle. Thus, we suggest that SCF^Dia2^ functions in transcriptional silencing in budding yeast, at least partly through ubiquitylation of Sir4.

It is not unprecedented for an E3 ubiquitin ligase to be involved in transcriptional silencing. In fact, several ubiquitin ligases and hydrolases have been shown to have roles in transcriptional silencing in budding yeast and other organisms. In *S. pombe*, the Rik1-Cul4 E3 ligase complex is important for the recruitment of the Clr4 histone methyltransferase for RNAi-mediated heterochromatin formation. Deletion of the Rik1-Cul4 complex results in silencing defects [49]–[51]. In budding yeast, Rtt101, a cullin protein of a ubiquitin E3 ligase complex, is important for telomeric silencing [52]. Rad6, involved in ubiquitylation of H2B, also impacts transcriptional silencing in budding yeast [53], [54]. Finally, Sir4 is known to interact with two ubiquitin hydrolases, Ubp3 and Dot4/Ubp10 [55], [56]. Ubp10 is proposed to regulate transcriptional silencing by deubiquitylating H2B [57]. How Ubp3 is involved in silencing is still unknown. Interestingly, loss of Upb3 results in improved silencing at telomeres and the *HM* loci (*HML*) [55], the opposite effect as that of loss of Dia2.

How does Sir4 ubiquitylation affect transcriptional silencing? Protein ubiquitylation, in general, is known to mediate two distinct functions. First, protein ubiquitylation marks proteins for degradation by the 26S proteasome. Second, protein ubiquitylation can also regulate protein-protein interactions [58], [59]. Interestingly, we did not detect significant changes in the steady-state levels of Sir4 in *dia2*Δ cells, suggesting that Sir4 ubiquitylation by SCF^Dia2^ is not likely involved in regulating the steady-state level of Sir4. However, we did observe a significant increase in Sir4 proteins at the *HMR* silent locus in *dia2Δ rtt106*Δ double mutant cells. It has been reported that β-catenin levels on chromatin, but not steady state levels, are regulated by ubiquitylation through a protein complex containing the histone acetyltransferase component TRRAP and Skp1 [60]. Therefore, it is possible that a SCF ubiquitin ligase can regulate protein levels on chromatin. We have shown previously that Rtt106 binds Sir4 [11]. While the functional significance of the Rtt106-Sir4 interaction is not clear, it is possible that this interaction regulates Sir4 binding to silent chromatin. This could explain why loss of Rtt106 leads to aberrant accumulation of Sir4 at silent chromatin in *dia2Δ rtt106*Δ mutant cells. Therefore, we suggest that Sir4 ubiquitylation by SCF^Dia2^ regulates Sir4 levels at chromatin, which in turn regulates silencing. Supporting this idea, we show that elevation of Sir4 levels using a centromere plasmid, while having no apparent effect on telomeric silencing in wild-type cells, reduces telomeric silencing in *dia2*Δ mutant cells.

It has been observed that heterochromatin proteins such as HP1 are dynamically regulated during mitotic cell division. For instance, in human cells, phosphorylation of serine 10 of histone H3 (H3S10ph) during mitosis reduces the binding affinity of HP1 towards H3K9me3, which propels the dissociation of HP1 from heterochromatin [42]. In *S. pombe*, dissociation of the sequence homolog of HP1, Swi6, from heterochromatin via H3S10ph results in transcription of siRNA during S phase. This, in turn, helps to maintain heterochromatin during S phase of the cell cycle [44]. These results highlight the fact that heterochromatin in *S. pombe* and mammalian cells is dynamically regulated during mitotic cell division. It was previously unknown whether silent chromatin in budding yeast was also regulated during S phase of the cell cycle. We observed that Sir4 binding to telomeric silent chromatin was significantly reduced as cells progressed from G1 to S and G2/M phase of the cell cycle. Concomitant with the reduction of Sir4 binding, the transcription of the telomere gene, *YFR057W*, increased when cells entered S phase. These results demonstrate that budding yeast silent chromatin is also dynamically regulated during S phase of the cell cycle. Because HP1 and histone modifications equivalent to H3K9me3 and H3S10ph are not present in budding yeast, we propose that perhaps SCF^Dia2^-mediated ubiquitylation of Sir4 serves as a mechanism to regulate Sir4 proteins and silent chromatin structure during the cell cycle. Further investigation is warranted to address such a role for the SCF^Dia2^ complex and Sir4 ubiquitylation.

In summary, our studies reveal a role for the SCF^Dia2^ E3 ligase in transcriptional silencing. In addition, we show that the SCF^Dia2^ E3 ligase binds and ubiquitylates Sir4. Furthermore, Dia2 is required for the regulation of Sir4 binding to chromatin during S phase of the cell cycle. These studies reveal a novel mechanism by which yeast silent chromatin is regulated during S phase of the cell cycle.

## Materials and Methods

### Yeast strains and plasmids

All yeast strains, except those for the SGA screen, were derived from W303 (*leu2-3, 112 ura3-1, his3-11, trp1-1, ade2-1 can1-100)* and constructed using standard methods and can be found listed in Table S1. The synthetic genetic array method to screen 4,700 viable yeast deletion mutants has been previously described in detail, along with the assay used to screen specifically for mutants that exhibit defects in *HMR* silencing [13], [21]. Detailed methods for the screen can be found in Text S1. Plasmids for Dia2 and Sir4 expression were constructed using standard methods in the vector, pRS313. Oligos used to construct the mutant Dia2 plasmids, as well as those used for analysis of mRNA expression and ChIP DNA using real-time PCR, are listed in Table S2.

### Silencing assays

Telomeric silencing was analyzed as described previously [13]. Briefly, yeast cells containing the *URA3* gene at the left arm of chromosome VII (*URA3-VIIL*) were plated onto the indicated media in a 10 fold series dilution with a starting OD_600_ of 6.0 for growth on 5-fluoroorotic acid (FOA) and OD_600_ 0.6 on media not containing FOA. Images were taken after four days of incubation at 30°C.

Assays for silencing at the *HMR* silent mating type locus were performed as described [13] in cells containing a GFP reporter integrated at the silent *HMR* locus. Cells were grown at 25°C to OD_600_ 0.6–0.8 and washed three times with synthetic complete (SC) –TRP media. The percentage of cells expressing GFP was determined using flow cytometry with the GFP populations in wild-type and *sir3*Δ cells as standards.

### Localization of Sir3-GFP and Sir4-GFP using fluorescence microscopy

Cells in which Sir3 or Sir4 were tagged with GFP were grown in YPD or selective media (SC-HIS) at 25°C and collected at OD_600_ 0.6–0.8. Cells were washed three times with SC-TRP media and analyzed using a Zeiss fluorescence microscope. Images were taken with z-stack images captured at every 0.3 µm, and one z-stack image was shown in Figure 4A. At least 100 cells of each genotype were counted from at least two independent experiments, and the percentage of cells exhibiting foci similar to wild-type cells was reported.

### Chromatin immunoprecipitation assay (ChIP)

ChIP assays were performed as described [11]. Cells were first fixed with 1% formaldehyde and then quenched with glycine. Cells were collected and homogenized using glass beads. Chromatin DNA was sheared to an average size of 0.5 to 1 kb by sonication and immunoprecipitated with specific antibodies against the protein of interest. The co-precipitated DNA was analyzed by real-time PCR using primers whose sequences are listed in Table S1. To analyze Sir4 levels during the cell cycle, cells were arrested for 3 hours with α-factor. After washing away α-factor with cold water three times, cells were released into fresh medium and collected at different time points for analysis of DNA content, gene expression and ChIP assays as described above.

### Ubiquitylation assays

Ubiquitylation assays were performed as described [41]. Briefly, Flag-ubiquitin, E1, E2, E3 (whole cell extracts prepared from cells expressing full length MYC-Dia2 or MYC-Dia2 F-boxΔ or purified MYC-Dia2 (full length and F-boxΔ). More details for both the *in vitro* and *in vivo* ubiquitylation assays can be found in Text S1.

## Supporting Information

Figure S1 *DIA2* and *RTT106* exhibit a synthetic interaction in transcriptional silencing at the *HMR* locus. Cells with the indicated genotype were collected for analysis of GFP expression via flow cytometry (A) and fluorescence microscopy (B). The percentage of cells expressing GFP was determined for both methods of detecting GFP expression. Experiments were performed as described in Figure 1A and 1B. Following detection of GFP expression using flow cytometry, the same cells were used to determine GFP expression by capturing images using a Zeiss fluorescence microscope. For microscope images, between 100–200 cells were counted for each strain analyzed, and the percentage of GFP expressing cells was calculated. Error bars represent the standard deviation (s.d) of values determined from two independent experiments, with two independent colonies used for determination of *dia2Δ HMR::GFP* and *dia2Δ rtt106Δ HMR::GFP* values. * represents a p-value <0.05 using the student's t-test in comparison with the result obtained for the W303 *HMR::GFP* strain. Other p-values and comparisons are as indicated.
(JPG)

Figure S2 The *dia2*Δ mutation genetically interacts with genes encoding histone chaperones in growth and response to DNA damaging agents. Epistasis analysis was carried out for *dia2*Δ with *cac1*Δ, *rtt106*Δ (A), *asf1Δ, hir1*Δ (B), and *cac1Δ rtt106*Δ (C) for growth and DNA damage sensitivity. Spot assays were performed in which cells were spotted in a 10 fold serial dilution onto regular growth media or media containing the indicated concentration of camptothecin (CPT) or methyl methanesulfonate (MMS). Images were taken following incubation for the indicated time. The results are summarized in Figure 2C.
(JPG)

Figure S3 Expression of Dia2 full length and mutant proteins are similar. (A) Whole cell extracts were collected from mutant *dia2*Δ cells transformed with empty vector or plasmid expressing full length Dia2 or mutant forms of Dia2 as indicated. Full length and mutant forms of Dia2 were detected via Western blot using antibodies against the MYC epitope, which was fused to the N-terminus of Dia2 (full length and mutant forms). * indicates non-specific band detected with the MYC antibody. Antibodies against Sir2 and PCNA were used as loading controls. (B) Quantification of the Western blot presented in A. Bands were quantified using ImageQuant. Dia2 (full length or mutant) expression was normalized against PCNA and Sir2. Then, data was analyzed as the expression of each sample over that of full length Dia2, and the average of values obtained using PCNA or Sir2 for normalization was reported.
(JPG)

Figure S4 Sir protein levels in *dia2*Δ cells are comparable to that of wild-type cells. (A–B) Sir3, Sir2 (A) and Sir4 (B) protein levels are similar in wild-type and *dia2*Δ mutant cells. Proteins in whole cell extracts collected from the indicated strains were analyzed by Western blot. Samples were loaded with two different amounts (1X or 2X). Sir3 and Sir4 were detected using antibodies against CBP (calmodulin binding peptide), and Sir2 was detected using an antibody against Sir2. W303 (No Tag) strain was used as a control for detection of Sir3-TAP or Sir4-TAP. Orc3 was used as a loading control.
(JPG)

Figure S5 Sir3 levels at silent chromatin loci are not significantly altered in *dia2*Δ and *dia2Δ rtt106*Δ cells. (A) Sir3 levels are not significantly altered at the *HMR* locus in *dia2*Δ and *dia2Δ rtt106*Δ cells. ChIP assay was performed in unsynchronized cells as described in Figure 5 using an antibody against endogenous Sir3. The upper panel describes the locations of the primers used to analyze regions at the *HMR* locus. (B–C) Sir3 levels are not significantly altered at telomere silent and active loci in *dia2*Δ and *dia2Δ rtt106*Δ cells. ChIP assays were performed in unsynchronized cells as described in Figure 5 using either antibody against endogenous Sir3 (B) or IgG sepharose to pull down Sir3-TAP (C). Real-time PCR was used to analyze Sir3 levels at silent and active regions of the telomere at the right arm of chromosome VI (upper panel of B). Data is presented as described in Figure 5D.
(JPG)

Figure S6 The expression of Sir4 or Sir2 is not altered in *dia2*Δ cells. (A–B) RNA was collected from cells of the indicated genotype, and Sir4 (A) and Sir2 (B) mRNA levels were analyzed as described in Figure 1C. Data is represented as the mean ± s.d. from two independent experiments.
(JPG)

Figure S7 The interaction between Sir4 and Sir2 is not altered to a detectable degree in *dia2*Δ cells. (A) Sir4-TAP was purified from cells of the indicated genotype (W303 was used as a No Tag control strain). Sir4 and Sir2 in soluble cell extracts (SCE) and immunoprecipitated (IP) samples were detected by Western blot using the indicated antibodies.
(JPG)

Table S1 List of strains used in this study.
(DOC)

Table S2 List of oligos used in the study. Oligos are listed 5′ to 3′, and their uses are shown. RNA: those primers used to analyze mRNA expression via real-time PCR of prepared cDNA; ChIP: those primers used to analyze ChIP DNA via real-time PCR; mutagenesis: those primers used to construct the various Dia2 mutants used in the study.
(DOC)

Text S1 Supporting Methods and Materials.
(DOC)
