# Supplementary figures and images for: The SCFDia2 Ubiquitin E3 Ligase Ubiquitylates Sir4 and Functions in Transcriptional Silencing
Source: PLoS Genet. 2012 Jul 26;8(7):e1002846. doi: 10.1371/journal.pgen.1002846 (PMC3405993; doi:10.1371/journal.pgen.1002846)

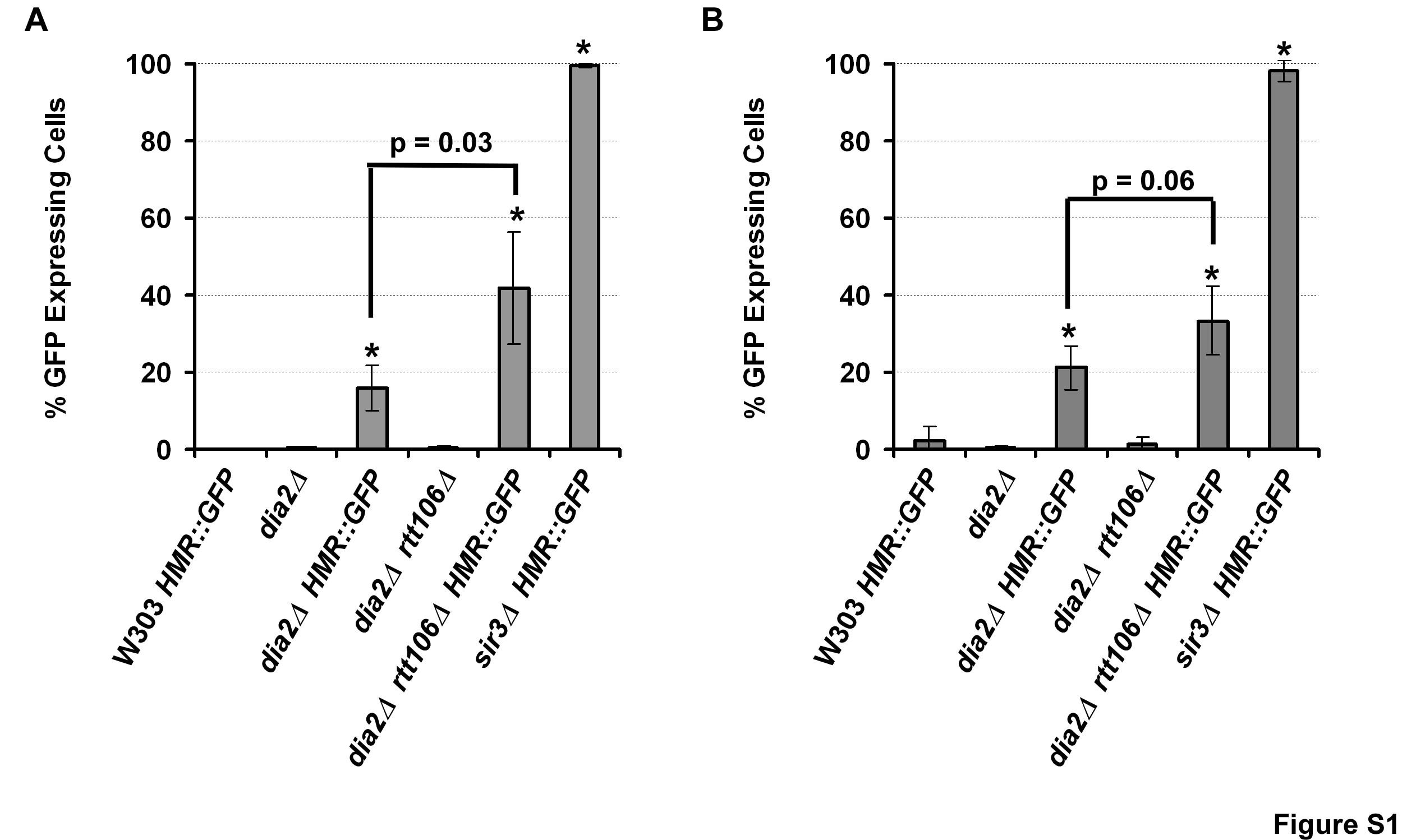

Supplement: Figure S1 — DIA2 and RTT106 exhibit a synthetic interaction in transcriptional silencing at the HMR locus. Cells with the indicated genotype were collected for analysis of GFP expression via flow cytometry (A) and fluorescence microscopy (B). The percentage of cells expressing GFP was determined for both methods of detecting GFP expression. Experiments were performed as described in Figure 1A and 1B. Following detection of GFP expression using flow cytometry, the same cells were used to determine GFP expression by capturing images using a Zeiss fluorescence microscope. For microscope images, between 100–200 cells were counted for each strain analyzed, and the percentage of GFP expressing cells was calculated. Error bars represent the standard deviation (s.d) of values determined from two independent experiments, with two independent colonies used for determination of dia2Δ HMR::GFP and dia2Δ rtt106Δ HMR::GFP values. * represents a p-value <0.05 using the student's t-test in comparison with the result obtained for the W303 HMR::GFP strain. Other p-values and comparisons are as indicated. (JPG) [file pgen.1002846.s001.jpg]

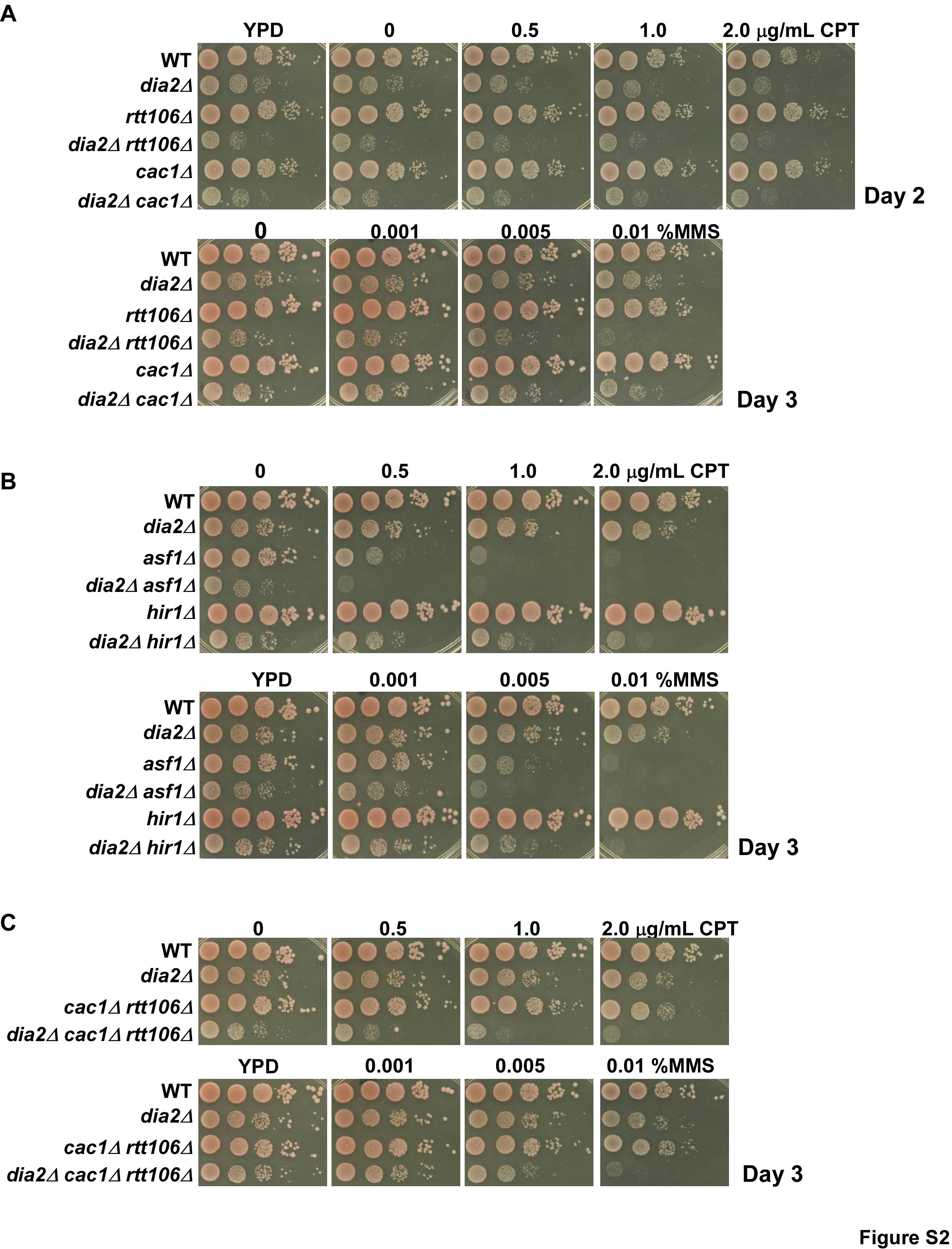

Supplement: Figure S2 — The dia2Δ mutation genetically interacts with genes encoding histone chaperones in growth and response to DNA damaging agents. Epistasis analysis was carried out for dia2Δ with cac1Δ, rtt106Δ (A), asf1Δ, hir1Δ (B), and cac1Δ rtt106Δ (C) for growth and DNA damage sensitivity. Spot assays were performed in which cells were spotted in a 10 fold serial dilution onto regular growth media or media containing the indicated concentration of camptothecin (CPT) or methyl methanesulfonate (MMS). Images were taken following incubation for the indicated time. The results are summarized in Figure 2C. (JPG) [file pgen.1002846.s002.jpg]

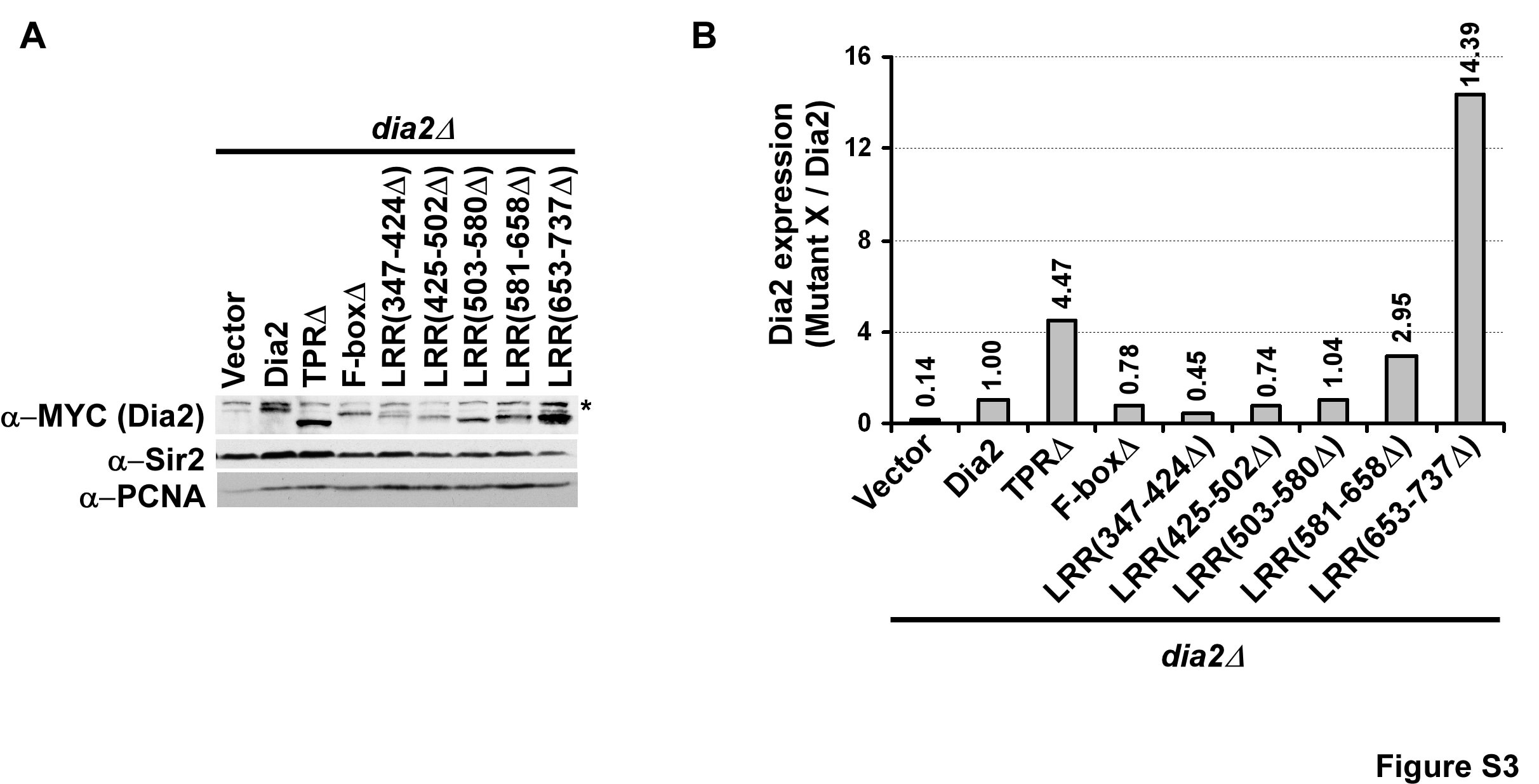

Supplement: Figure S3 — Expression of Dia2 full length and mutant proteins are similar. (A) Whole cell extracts were collected from mutant dia2Δ cells transformed with empty vector or plasmid expressing full length Dia2 or mutant forms of Dia2 as indicated. Full length and mutant forms of Dia2 were detected via Western blot using antibodies against the MYC epitope, which was fused to the N-terminus of Dia2 (full length and mutant forms). * indicates non-specific band detected with the MYC antibody. Antibodies against Sir2 and PCNA were used as loading controls. (B) Quantification of the Western blot presented in A. Bands were quantified using ImageQuant. Dia2 (full length or mutant) expression was normalized against PCNA and Sir2. Then, data was analyzed as the expression of each sample over that of full length Dia2, and the average of values obtained using PCNA or Sir2 for normalization was reported. (JPG) [file pgen.1002846.s003.jpg]

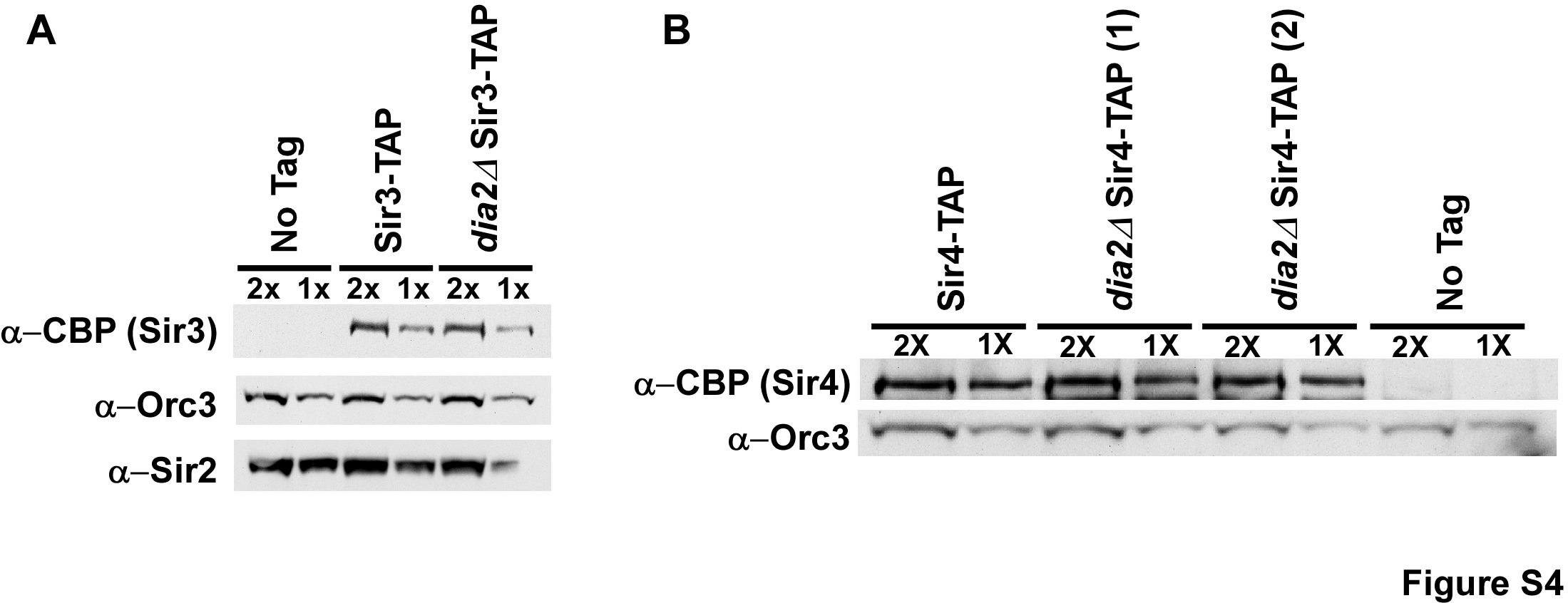

Supplement: Figure S4 — Sir protein levels in dia2Δ cells are comparable to that of wild-type cells. (A–B) Sir3, Sir2 (A) and Sir4 (B) protein levels are similar in wild-type and dia2Δ mutant cells. Proteins in whole cell extracts collected from the indicated strains were analyzed by Western blot. Samples were loaded with two different amounts (1X or 2X). Sir3 and Sir4 were detected using antibodies against CBP (calmodulin binding peptide), and Sir2 was detected using an antibody against Sir2. W303 (No Tag) strain was used as a control for detection of Sir3-TAP or Sir4-TAP. Orc3 was used as a loading control. (JPG) [file pgen.1002846.s004.jpg]

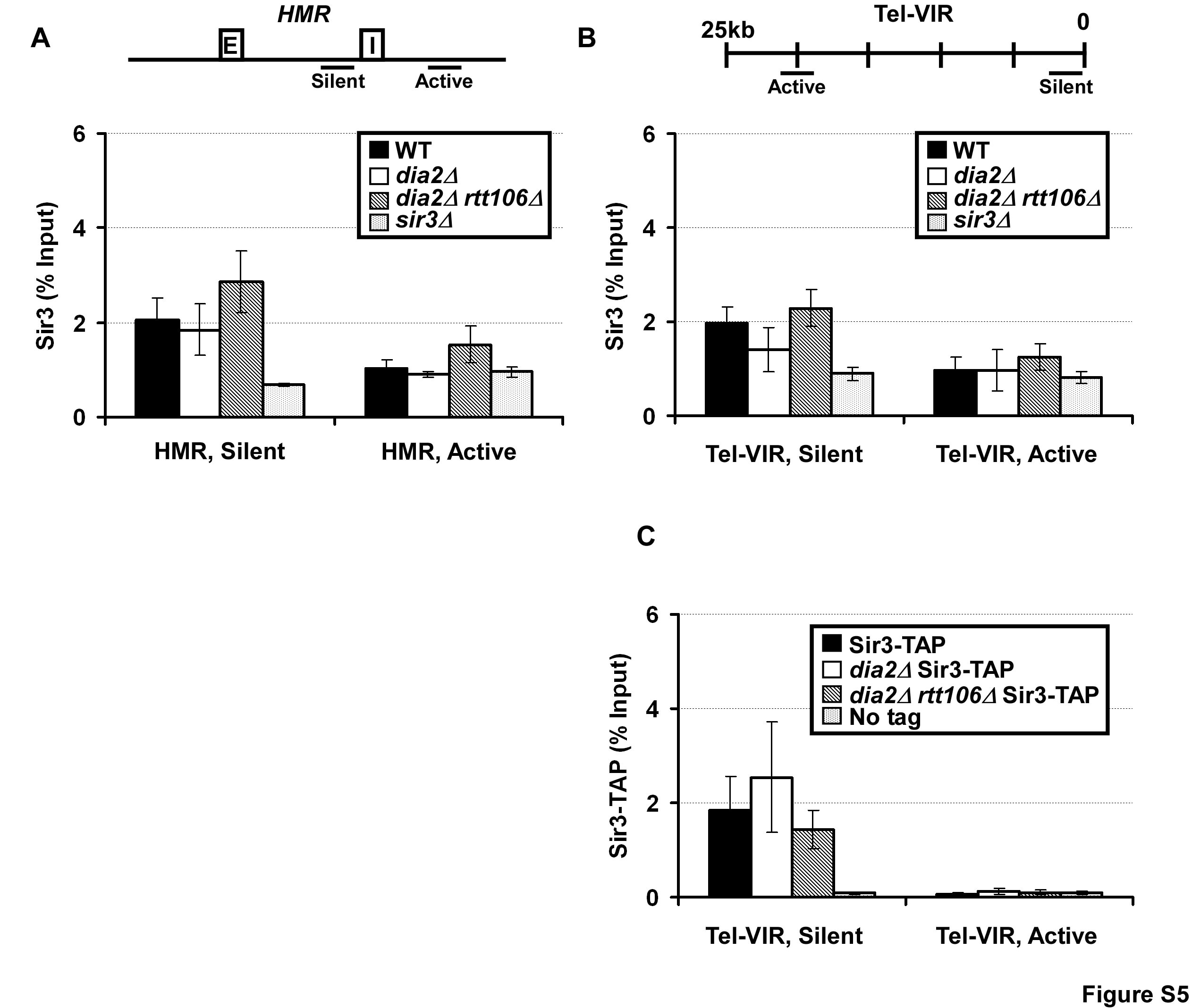

Supplement: Figure S5 — Sir3 levels at silent chromatin loci are not significantly altered in dia2Δ and dia2Δ rtt106Δ cells. (A) Sir3 levels are not significantly altered at the HMR locus in dia2Δ and dia2Δ rtt106Δ cells. ChIP assay was performed in unsynchronized cells as described in Figure 5 using an antibody against endogenous Sir3. The upper panel describes the locations of the primers used to analyze regions at the HMR locus. (B–C) Sir3 levels are not significantly altered at telomere silent and active loci in dia2Δ and dia2Δ rtt106Δ cells. ChIP assays were performed in unsynchronized cells as described in Figure 5 using either antibody against endogenous Sir3 (B) or IgG sepharose to pull down Sir3-TAP (C). Real-time PCR was used to analyze Sir3 levels at silent and active regions of the telomere at the right arm of chromosome VI (upper panel of B). Data is presented as described in Figure 5D. (JPG) [file pgen.1002846.s005.jpg]

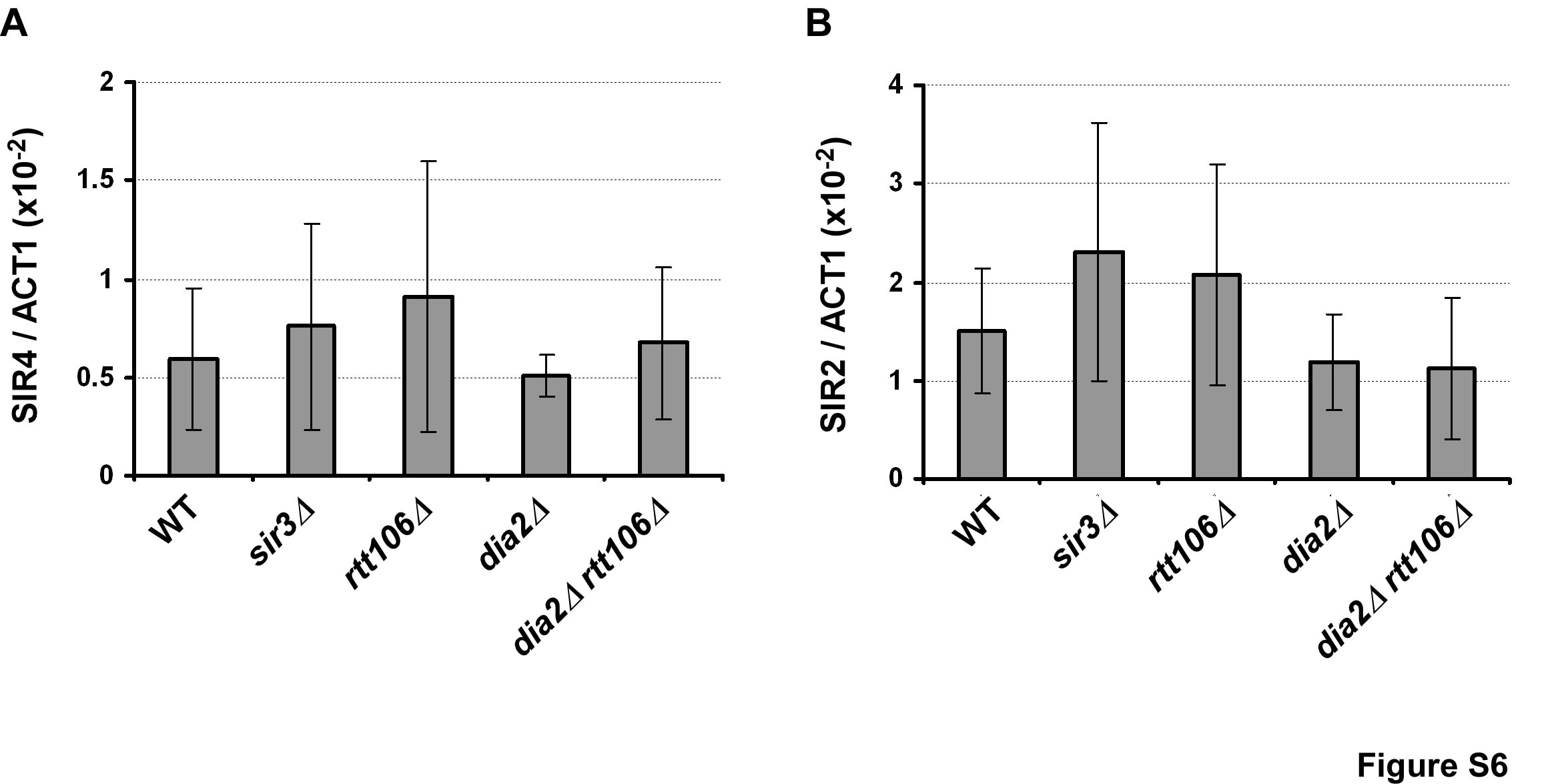

Supplement: Figure S6 — The expression of Sir4 or Sir2 is not altered in dia2Δ cells. (A–B) RNA was collected from cells of the indicated genotype, and Sir4 (A) and Sir2 (B) mRNA levels were analyzed as described in Figure 1C. Data is represented as the mean ± s.d. from two independent experiments. (JPG) [file pgen.1002846.s006.jpg]

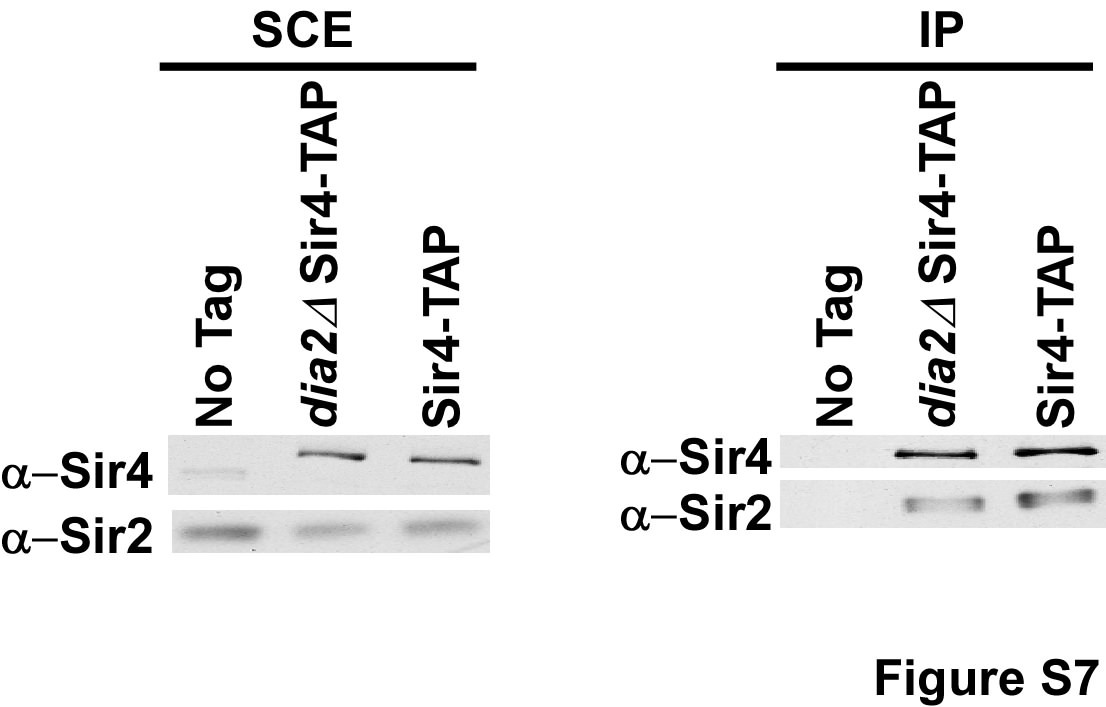

Supplement: Figure S7 — The interaction between Sir4 and Sir2 is not altered to a detectable degree in dia2Δ cells. (A) Sir4-TAP was purified from cells of the indicated genotype (W303 was used as a No Tag control strain). Sir4 and Sir2 in soluble cell extracts (SCE) and immunoprecipitated (IP) samples were detected by Western blot using the indicated antibodies. (JPG) [file pgen.1002846.s007.jpg]
