# Supplementary material for: The SCFDia2 Ubiquitin E3 Ligase Ubiquitylates Sir4 and Functions in Transcriptional Silencing
Source: PLoS Genet. 2012 Jul 26;8(7):e1002846. doi: 10.1371/journal.pgen.1002846 (PMC3405993; doi:10.1371/journal.pgen.1002846)
Supplement: Table S1 — List of strains used in this study. (DOC) [file pgen.1002846.s008.doc]

**Table S1**

| **Name** | **Genotype** | **Reference** |
| --- | --- | --- |
| ZGY1589 | *MATa* *can1Δ*::*MFA1pr-HIS3 lyp1Δ his-1,* *leu2-0, met15-0 LYS2^+^* *hmr::GFP-URA3, rad52::rad52-RFP::hph, rtt106Δ::natR* | This study |
| ZGY449 | *MATa leu2-3, 112 ura3-1 his3-11,15, trp1-1, ade2-1, can1-100,* *hmr::GFP-URA3* | [2] |
| ZGY476 | *MATa leu2-3, 112 ura3-1 his3-11,15, trp1-1, ade2-1, can1-100,* *hmr::GFP-URA3, rtt106Δ::natR* | [2] |
| ZGY512 | *MATa leu2-3, 112 ura3-1 his3-11,15, trp1-1, ade2-1, can1-100,* *hmr::GFP-URA3,sir3Δ::TRP1* | [2] |
| ZGY1540 | *MATa leu2-3, 112 ura3-1 his3-11,15, trp1-1, ade2-1, can1-100,* *hmr::GFP-URA3, dia2Δ::kanMX6* | This study |
| ZGY1597 | *MATa leu2-3, 112 ura3-1 his3-11,15, trp1-1, ade2-1, can1-100,* *hmr::GFP-URA3, dia2Δ::kanMX6, rtt106Δ::natR* | This study |
| ZGY451 | *MATa leu2-3, 112 ura3-1 his3-11,15, trp1-1, ade2-1, can1-100,* *hmr::GFP-URA3, hir1Δ::kanMX6* | [2] |
| ZGY1631 | *MATa leu2-3, 112 ura3-1 his3-11,15, trp1-1, ade2-1, can1-100,* *hmr::GFP-URA3, hir1Δ::kanMX6, dia2Δ::kanMX6* | This study |
| ZGY452 | *MATa leu2-3, 112 ura3-1 his3-11,15, trp1-1, ade2-1, can1-100,* *hmr::GFP-URA3, asf1Δ::kanMX6* | [2] |
| ZGY1638 | *MATa leu2-3, 112 ura3-1 his3-11,15, trp1-1, ade2-1, can1-100,* *hmr::GFP-URA3, asf1Δ::natR, dia2Δ::kanMX6* | This study |
| ZGY450 | *MATa leu2-3, 112 ura3-1 his3-11,15, trp1-1, ade2-1, can1-100,* *hmr::GFP-URA3, cac1Δ::LEU2* | [2] |
| ZGY1544 | *MATa leu2-3, 112 ura3-1 his3-11,15, trp1-1, ade2-1, can1-100,* *hmr::GFP-URA3, cac1Δ::LEU2, dia2Δ::kanMX6* | This study |
| YB542 | *MATα leu2-3, 112 ura3-1 his3-11,15, trp1-1, ade2-1, can1-100,* *URA3-VIIL* | [2] |
| ZGY1705 | *MATa leu2-3, 112 ura3-1 his3-11,15, trp1-1, ade2-1, can1-100,* *dia2Δ::kanMX6, URA3-VIIL* | This study |
| ZGY602 | *MATa leu2-3, 112 ura3-1 his3-11,15, trp1-1, ade2-1, can1-100,* *rtt106Δ::natRMX, URA3-VIIL* | [2] |
| ZGY1704 | *MATα leu2-3, 112 ura3-1 his3-11,15, trp1-1, ade2-1, can1-100,* *rtt106Δ::natR, dia2Δ::kanMX6, URA3-VIIL* | This study |
| ZGY739 | *MATa leu2-3, 112 ura3-1 his3-11,15, trp1-1, ade2-1, can1-100,* *sir3Δ::kanMX6, URA3-VIIL* | This study |
| ZGY337 | *MATa leu2-3, 112 ura3-1 his3-11,15, trp1-1, ade2-1, can1-100, Sir3-GFP::URA3* | [7] |
| ZGY339 | *MATa leu2-3, 112 ura3-1 his3-11,15, trp1-1, ade2-1, can1-100, GFP-Sir4::URA3* | [7] |
| ZGY1965 | *MATa leu2-3, 112 ura3-1 his3-11,15, trp1-1, ade2-1, can1-100, dia2Δ::kanMX6 Sir3-GFP::URA3* | This study |
| ZGY2150 | *MATa leu2-3, 112 ura3-1 his3-11,15, trp1-1, ade2-1, can1-100, dia2Δ::kanMX6, GFP-Sir4::URA3* | This study |
| W303-1B | *MATα leu2-3, 112 ura3-1 his3-11,15, trp1-1, ade2-1, can1-100* | [8] |
| ZGY1519 | *MATα leu2-3, 112 ura3-1 his3-11,15, trp1-1, ade2-1, can1-100, dia2Δ::KanMX6* | This study |
| ZGY766 | *MATα leu2-3, 112 ura3-1 his3-11,15, trp1-1, ade2-1, can1-100, sir3Δ::KanMX6* | [7] |
| ZGY853 | *MATa leu2-3, 112 ura3-1 his3-11,15, trp1-1, ade2-1, can1-100, hht1-hhf1::leu2,hht2-hhf2::kanMX6, HHT2 (K56R)-HHF2/*pRS313 | [9] |
| ZGY1077 | *MATa leu2-3, 112 ura3-1 his3-11,15, trp1-1, ade2-1, can1-100, hht1-hhf1::leu2,hht2-hhf2::kanMX6, HHT1 (K9,14, 18, 23, 27R)-HHT2/*pRS414 | [9] |
| ZGY1921 | *MATa leu2-3, 112 ura3-1 his3-11,15, trp1-1, ade2-1, can1-100, hht1-hhf1::leu2,hht2-hhf2::kanMX6, HHT1-HHT2(K16R)/*pRS313 | This study |
| ZGY720 | *MATa leu2-3, 112 ura3-1 his3-11,15, trp1-1, ade2-1, can1-100, rtt106Δ::kanMX6, Sir3-GFP::URA3* | [7] |
| ZGY722 | *MATa leu2-3, 112 ura3-1 his3-11,15, trp1-1, ade2-1, can1-100, rtt106Δ::kanMX6, GFP-Sir4::URA3* | [7] |
| ZGY2238 | *MATa leu2-3, 112 ura3-1 his3-11,15, trp1-1, ade2-1, can1-100, dia2Δ::kanMX6, rtt106Δ::natR, Sir3-GFP::URA3* | This study |
| ZGY2240 | *MATa leu2-3, 112 ura3-1 his3-11,15, trp1-1, ade2-1, can1-100, dia2Δ::kanMX6, rtt106Δ::natR, GFP-Sir4::URA3* | This study |
| ZGY484 | *MATα leu2-3, 112 ura3-1 his3-11,15, trp1-1, ade2-1, can1-100, rtt106Δ::natR* | [9] |
| ZGY2234 | *MATα leu2-3, 112 ura3-1 his3-11,15, trp1-1, ade2-1, can1-100, rtt106Δ::natR, dia2Δ::kanMX6* | This study |
| ZGY1704 | *MATα leu2-3, 112 ura3-1 his3-11,15, trp1-1, ade2-1, can1-100, rtt106Δ::natR, dia2Δ::kanMX6, URA3-VIIL* | This study |
| ZGY471 | *MATa leu2-3, 112 ura3-1 his3-11,15, trp1-1, ade2-1, can1-100, cac1Δ::LEU2, rtt106Δ::natR, URA3-VIIL* | [2] |
| ZGY812 | *MATα leu2-3, 112 ura3-1 his3-11,15, trp1-1, ade2-1, can1-100, cac1Δ::LEU2, URA3-VIIL* | [2] |
| ZGY1594 | *MATα leu2-3, 112 ura3-1 his3-11,15, trp1-1, ade2-1, can1-100, cac1Δ::LEU2, dia2Δ::kanMX6, URA3-VIIL* | This study |
| ZGY1596 | *MATα leu2-3, 112 ura3-1 his3-11,15, trp1-1, ade2-1, can1-100, cac1Δ::LEU2, rtt106Δ::natR, dia2Δ::kanMX6, URA3-VIIL* | This study |
| ZGY645 | *MATα leu2-3, 112 ura3-1 his3-11,15, trp1-1, ade2-1, can1-100, asf1Δ::natR, URA3-VIIL* | This study |
| ZGY1633 | *MATα leu2-3, 112 ura3-1 his3-11,15, trp1-1, ade2-1, can1-100, asf1Δ::natR, dia2Δ::kanMX6, URA3-VIIL* | This study |
| ZGY574 | *MATα leu2-3, 112 ura3-1 his3-11,15, trp1-1, ade2-1, can1-100, cac1Δ::LEU2* | [10] |
| ZGY1543 | *MATα leu2-3, 112 ura3-1 his3-11,15, trp1-1, ade2-1, can1-100, cac1Δ::LEU2, dia2Δ::kanMX6* | This study |
| ZGY764 | *MATα leu2-3, 112 ura3-1 his3-11,15, trp1-1, ade2-1, can1-100, cac1Δ::LEU2, rtt106Δ::natR* | This study |
| ZGY684 | *MATα leu2-3, 112 ura3-1 his3-11,15, trp1-1, ade2-1, can1-100, hir1Δ::kanMX6* | This study |
| ZGY1547 | *MATα leu2-3, 112 ura3-1 his3-11,15, trp1-1, ade2-1, can1-100, cac1Δ::LEU2, rtt106Δ::natR, dia2Δ::kanMX6* | This study |
| ZGY1926 | *MATα leu2-3, 112 ura3-1 his3-11,15, trp1-1, ade2-1, can1-100, ctf4Δ::kanMX6, URA3-VIIL* | This study |
| ZGY1927 | *MATα leu2-3, 112 ura3-1 his3-11,15, trp1-1, ade2-1, can1-100, mrc1Δ::kanMX6, URA3-VIIL* | This study |
| ZGY2227 | *MATα leu2-3, 112 ura3-1 his3-11,15, trp1-1, ade2-1, can1-100, ctf4Δ::kanMX6, dia2Δ::kanMX6, URA3-VIIL* | This study |
| ZGY2229 | *MATα leu2-3, 112 ura3-1 his3-11,15, trp1-1, ade2-1, can1-100, mrc1Δ::kanMX6, dia2Δ::kanMX6, URA3-VIIL* | This study |
| DMY1737 | *MATα ura3-52, trp1, lys2-801, leu2 Δ1, pep4Δ::HIS3, prb1Δ1.6R, can1, SIR3::TAP::TRP1* | [5] |
| DMY1704 | *MATα ura3-52, trp1, lys2-801, leu2 Δ1, pep4Δ::HIS3, prb1Δ1.6R, can1, SIR4::TAP::TRP1* | [5] |
| ZGY2129 | *MATα ura3-52, trp1, lys2-801, leu2 Δ1, pep4Δ::HIS3, prb1Δ1.6R, can1, SIR3::TAP::TRP1, dia2Δ::kanMX6* | This study |
| ZGY2130 | *MATα ura3-52, trp1, lys2-801, leu2 Δ1, pep4Δ::HIS3, prb1Δ1.6R, can1, SIR4::TAP::TRP1, dia2Δ::kanMX6* | This study |
| ZGY2223 | *MATα ura3-52, trp1, lys2-801, leu2 Δ1, pep4Δ::HIS3, prb1Δ1.6R, can1, SIR4::TAP::TRP1, dia2Δ::kanMX6, rtt106Δ::natR* | This study |
| ZGY1987 | *MATa leu2-3, 112 ura3-1 his3-11,15, trp1-1, ade2-1, can1-100, hht1-hhf1::leu2,hht2-hhf2::kanMX6, HHT2 (K56R)-HHF2/*pRS313, *dia2Δ::kanMX6* | This study |
| ZGY1989 | *MATa leu2-3, 112 ura3-1 his3-11,15, trp1-1, ade2-1, can1-100, hht1-hhf1::leu2,hht2-hhf2::kanMX6, HHT2-HHF2/*yCP50, *dia2Δ::kanMX6* | This study |
| ZGY1000 | *MATa leu2-3, 112 ura3-1 his3-11,15, trp1-1, ade2-1, can1-100, hht1-hhf1::leu2,hht2-hhf2::kanMX6, HHT2-HHF2/*yCP50 | This study |
| ZGY1988 | *MATa leu2-3, 112 ura3-1 his3-11,15, trp1-1, ade2-1, can1-100, hht1-hhf1::leu2,hht2-hhf2::kanMX6, HHT1 (K9,14, 18, 23, 27R)-HHT2/*pRS414*, dia2Δ::kanMX6* | This study |
| ZGY1922 | *MATa leu2-3, 112 ura3-1 his3-11,15, trp1-1, ade2-1, can1-100, hht1-hhf1::leu2,hht2-hhf2::kanMX6, HHT1-HHT2(K16R)/*pRS313, *dia2Δ::kanMX6* | This study |
| ZGY1402 | *MATa leu2-3, 112 ura3-1 his3-11,15, trp1-1, ade2-1, can1-100, hht1-hhf1::leu2,hht2-hhf2::kanMX6, HHT2 (K27R)-HHF2/*pRS313 | [4] |
| ZGY1925 | *MATa leu2-3, 112 ura3-1 his3-11,15, trp1-1, ade2-1, can1-100, hht1-hhf1::leu2,hht2-hhf2::kanMX6, HHT2 (K27R)-HHF2/*pRS313, *dia2Δ::kanMX6* | This study |
| ZGY1087 | *MATa leu2-3, 112 ura3-1 his3-11,15, trp1-1, ade2-1, can1-100, hht1-hhf1::leu2,hht2-hhf2::kanMX6, HHT2-HHF2 (5,12R)/*pRS414 | [9] |
| ZGY1864 | *MATa leu2-3, 112 ura3-1 his3-11,15, trp1-1, ade2-1, can1-100, hht1-hhf1::leu2,hht2-hhf2::kanMX6, HHT2-HHF2 (5,12R)/*pRS414, *dia2Δ::kanMX6* | This study |
| ZGY1698 | *MATa leu2-3, 112 ura3-1 his3-11,15, trp1-1, ade2-1, can1-100, hht1-hhf1::leu2,hht2-hhf2::kanMX6, HHT2-HHF2 (8R)/*pRS313, *URA3-VIIL* | [10] |
| ZGY1862 | *MATa leu2-3, 112 ura3-1 his3-11,15, trp1-1, ade2-1, can1-100, hht1-hhf1::leu2,hht2-hhf2::kanMX6, HHT2-HHF2 (8R)/*pRS313, *dia2Δ::kanMX6* | This study |
| ZGY1861 | *MATa leu2-3, 112 ura3-1 his3-11,15, trp1-1, ade2-1, can1-100, hht1-hhf1::leu2,hht2-hhf2::kanMX6, HHT2-HHF2 (91R)/*pRS313, | This study |
| ZGY1860 | *MATa leu2-3, 112 ura3-1 his3-11,15, trp1-1, ade2-1, can1-100, hht1-hhf1::leu2,hht2-hhf2::kanMX6, HHT2-HHF2 (91R)/*pRS313, *dia2Δ::kanMX6* | This study |
| ZGY1452 | *MATa leu2-3, 112 ura3-1 his3-11,15, trp1-1, ade2-1, can1-100, hht1-hhf1::leu2,hht2-hhf2::kanMX6, HHT2(K14,27R)-HHF2/*pRS414, *URA3-VIIL* | This study |
| ZGY1859 | *MATa leu2-3, 112 ura3-1 his3-11,15, trp1-1, ade2-1, can1-100, hht1-hhf1::leu2,hht2-hhf2::kanMX6, HHT2(K14,27R)-HHF2/*pRS414, *dia2Δ::kanMX6* | This study |
| ZGY1094 | *MATa leu2-3, 112 ura3-1 his3-11,15, trp1-1, ade2-1, can1-100, hht1-hhf1::leu2,hht2-hhf2::kanMX6, HHT2-HHF2 (8,16R)/*pWZ414 | This study |
| ZGY1863 | *MATa leu2-3, 112 ura3-1 his3-11,15, trp1-1, ade2-1, can1-100, hht1-hhf1::leu2,hht2-hhf2::kanMX6, HHT2-HHF2 (8,16R)/*pWZ414, *dia2Δ::kanMX6* | This study |
| ZGY1919 | *MATa leu2-3, 112 ura3-1 his3-11,15, trp1-1, ade2-1, can1-100, hht1-hhf1::leu2,hht2-hhf2::kanMX6, HHT2(14R)-HHF2/*pRS414 | This study |
| ZGY1923 | *MATa leu2-3, 112 ura3-1 his3-11,15, trp1-1, ade2-1, can1-100, hht1-hhf1::leu2,hht2-hhf2::kanMX6, HHT2(14R)-HHF2/*pRS414, *dia2Δ::kanMX6* | This study |
| ZGY1920 | *MATa leu2-3, 112 ura3-1 his3-11,15, trp1-1, ade2-1, can1-100, hht1-hhf1::leu2,hht2-hhf2::kanMX6, HHT2(9R)-HHF2/*pRS414 | This study |
| ZGY1924 | *MATa leu2-3, 112 ura3-1 his3-11,15, trp1-1, ade2-1, can1-100, hht1-hhf1::leu2,hht2-hhf2::kanMX6, HHT2(9R)-HHF2/*pRS414, *dia2Δ::kanMX6* | This study |
| ZGY1450 | *MATa leu2-3, 112 ura3-1 his3-11,15, trp1-1, ade2-1, can1-100, hht1-hhf1::leu2,hht2-hhf2::kanMX6, HHT2(9,14R)-HHF2/*pRS314, *URA3-VIIL* | This study |
| ZGY1858 | *MATa leu2-3, 112 ura3-1 his3-11,15, trp1-1, ade2-1, can1-100, hht1-hhf1::leu2,hht2-hhf2::kanMX6, HHT2(9,14R)-HHF2/*pRS414, *dia2Δ::kanMX6* | This study |
| ZGY1451 | *MATa leu2-3, 112 ura3-1 his3-11,15, trp1-1, ade2-1, can1-100, hht1-hhf1::leu2,hht2-hhf2::kanMX6, HHT2(9,27R)-HHF2/*pRS414, *URA3-VIIL* | This study |
| ZGY1857 | *MATa leu2-3, 112 ura3-1 his3-11,15, trp1-1, ade2-1, can1-100, hht1-hhf1::leu2,hht2-hhf2::kanMX6, HHT2(9,27R)-HHF2/*pRS414, *dia2Δ::kanMX6* | This study |
| ZGY2151 | *MATa leu2-3, 112 ura3-1 his3-11,15, trp1-1, ade2-1, can1-100,* *dia2Δ::kanMX6-9MYC-Dia2::URA3* | This study |
| ZGY2153 | *MATa leu2-3, 112 ura3-1 his3-11,15, trp1-1, ade2-1, can1-100,* *dia2Δ::kanMX6-9MYC-Dia2-FboxΔ::URA3* | This study |
