# Supplementary material for: The SCFDia2 Ubiquitin E3 Ligase Ubiquitylates Sir4 and Functions in Transcriptional Silencing
Source: PLoS Genet. 2012 Jul 26;8(7):e1002846. doi: 10.1371/journal.pgen.1002846 (PMC3405993; doi:10.1371/journal.pgen.1002846)
Supplement: Table S2 — List of oligos used in the study. Oligos are listed 5′ to 3′, and their uses are shown. RNA: those primers used to analyze mRNA expression via real-time PCR of prepared cDNA; ChIP: those primers used to analyze ChIP DNA via real-time PCR; mutagenesis: those primers used to construct the various Dia2 mutants used in the study. (DOC) [file pgen.1002846.s009.doc]

**Table S2**

| **Name** | **Sequence** | **Purpose** |
| --- | --- | --- |
| YFR057W f | 5’-CTAGTGTCTATAGTAAGTGCTCGG-3’ | RNA |
| YFR057W r1 | 5’-GGTATATTGCCACGCAAAGAAAGG-3’ | RNA |
| Sir4 f | 5’-TTCATACCTCACCTCATCAACC-3’ | RNA |
| Sir4 r | 5’-TTGCCAAGTTTATGCCTAGAATC-3’ | RNA |
| Sir2 f | 5’-CCCTCCTTCACTAAACGAGAGTC-3’ | RNA |
| Sir2 r | 5’-AATGAAGTTGAAACACCTGCAC-3’ | RNA |
| a1 f | 5’-GGCGGAAAACATAAACAGAACTCTG-3’ | RNA |
| a1 r | 5’- Ccgtgcttggggtgatattgatg-3’ | RNA |
| ACT-1 | 5’- GGAAACGTAGAAGGCTGGAACGTT-3’ | RNA |
| ACT-2 | 5’- ACAACGAATTGAGAGTTGCCCCAG-3’ | RNA |
| VI-R 0.77-2 | 5’-GGACAGATCCTTTCGCATTCCTAC-3’ | ChIP |
| VI-R 0.77-3 | 5’-GCGCCTAGTGCAACTAGTGCATAT-3’ | ChIP |
| VI-R20-1 | 5’-GGATTGACGGGTAACCCTAAAAGG-3’ | ChIP |
| VI-R20-2 | 5’-GTTGTCATGGCCAATGACCACGAT-3’ | ChIP |
| hmr 7a f | 5’-AAGGATACGGTTTGAATCAATTTAC-3’ | ChIP |
| hmr-7 | 5’-AACTAAAAGAAAAACCCGACTATGC-3’ | ChIP |
| hmr-20 | 5’-CGAGTTCTTCTATATCCGGTGTAC-3’ | ChIP |
| hmr-21 | 5’-ACCAGGATCTTTTATCTGATAAGC-3’ | ChIP |
| Dia2 TPR (33-158) f | 5’-ATAGATTCTACCGTTCTGAAAGCAGATAGCAATCATCTAAGACAACAA-3’ | mutagenesis |
| Dia2 TPR (33-158) r | 5’-TTGTTGTCTTAGATGATTGCTATCTGCTTTCAGAACGGTAGAATCTAT-3’ | mutagenesis |
| Dia2 fbox (218-267) f | 5’-GGTAGTACCAAGAAAACTTTGGCACCAATAAATTTT-3’ | mutagenesis |
| Dia2 fbox (218-267) r | 5’-AAAATTTATTGGTGCCAAAGTTTTCTTGGTACTACC-3’ | mutagenesis |
| Dia2 LRR (347-737) f | 5’-CAAATCTTTAAGTTAATGGTTAGATTTGGAATTAATTCATATTCATAC-3’ | mutagenesis |
| Dia2 LRR (347-737) r | 5’-GTATGAATATGAATTAATTCCAAATCTAACCATTAACTTAAAGATTTG-3’ | mutagenesis |
| Dia2 LRR1 (347-424) f | 5’-CAAATCTTTAAGTTAATGGTTAGAGATGAACAGGGAATAGTAGAAGAA-3’ | mutagenesis |
| Dia2 LRR1 (347-424) r | 5’-TTCTTCTACTATTCCCTGTTCATCTCTAACCATTAACTTAAAGATTTG-3’ | mutagenesis |
| Dia2 LRR2 (425-502) f | 5’-CAGATATCCACTGCGGATAATGAAGATAACGACAACTGTGAACTC-3’ | mutagenesis |
| Dia2 LRR2 (425-502) r | 5’-GAGTTCACAGTTGTCGTTATCTTCATTATCCGCAGTGGATATCTG-3’ | mutagenesis |
| Dia2 LRR3 (503-580) f | 5’-CCAGACCTAAAAGAACTATGGATAGCGTTGACTAGATTATGCGAGCAG-3’ | mutagenesis |
| Dia2 LRR3 (503-580) r | 5’-CTGCTCGCATAATCTAGTCAACGCTATCCATAGTTCTTTTAGGTCTGG-3’ | mutagenesis |
| Dia2 LRR4 (581-658) f | 5’-ATGGGGACATCGATAAGTGGTTCTTTGGAAAATTTAAAGAGGCTTGAC-3’ | mutagenesis |
| Dia2 LRR4 (581-658) r | 5’-GTCAAGCCTCTTTAAATTTTCCAAAGAACCACTTATCGATGTCCCCAT-3’ | mutagenesis |
| Dia2 LRR5 (653-737) f | 5’-AATGATAGTACGATGAAGTCATTCGGAATTAATTCATATTCATAC-3’ | mutagenesis |
| Dia2 LRR5 (653-737) r | 5’-GTATGAATATGAATTAATTCCGAATGACTTCATCGTACTATCATT-3’ | mutagenesis |
