# Supplementary material for: The SCFDia2 Ubiquitin E3 Ligase Ubiquitylates Sir4 and Functions in Transcriptional Silencing
Source: PLoS Genet. 2012 Jul 26;8(7):e1002846. doi: 10.1371/journal.pgen.1002846 (PMC3405993; doi:10.1371/journal.pgen.1002846)
Supplement: Text S1 — Supporting Methods and Materials. (DOC) [file pgen.1002846.s010.doc]

**Text S1: Supporting Methods and Materials**

**Synthetic Genetic Array (SGA)**

SGA analysis was performed as previously described [1,2]. The bait strain, *rtt106∆, HMR::GFP* (BY4741 background) was crossed with ~4700 viable single mutants. Following selection of haploid double mutants containing the *HMR::GFP* cassette, cells were transferred to selective media in 384 plates for growth overnight at 30˚C. The following day, cells were analyzed for GFP expression by flow cytometry via high throughput analysis in the BD LSR II (BD Biosciences) machine within the Mayo Clinic Flow Cytometry and Optical Morphology core facility. Parameters were set prior to data collection with the bait and *rtt106∆ sir1∆* strains as standards. Following collection, flow cytometry profiles were analyzed for those double mutants that had at least 10% of cells expressing GFP and a clear rightward shift in the profile, indicating GFP expression. The GFP expression phenotype was confirmed using the method described in Figure 1A with cells grown in selective media.

**mRNA extraction and analysis**

RNA was collected from yeast cells using standard methods [3]. Briefly, log phase cells were collected and harvested. The cell pellet was resuspended in 400 μL of AE buffer (50 mM NaAcetate, pH 5.3, 10 mM EDTA) and then 40 μL of 10% SDS was added. After vortexing, an equal volume of phenol was added, and the mixture was incubated at 65˚C for four minutes. Following incubation, the mixture was rapidly chilled using a dry ice/ethanol bath and then centrifuged for 2 minutes at maximum speed. The aqueous phase was transferred to a fresh tube and extracted with phenol/chloroform for five minutes at room temperature. RNA was then purified using ethanol precipitation and resuspended in 40 μL of water. Isolated RNA was then reverse transcribed using M-MLV reverse transcriptase (Superscript III Reverse Transcriptase, Invitrogen) according to the manufacturer’s protocol. cDNA was analyzed using a BioRad real time PCR machine. Expression for each gene analyzed was normalized against that of *ACT1*.

**Yeast whole cell extraction**

Cells were grown to OD_600_ 0.8-1.0 and harvested at 3000rpm for 5 minutes. The cell pellets were washed with cold H_2_O and then boiled for 3 minutes. Protein extraction from yeast cells was performed using the glass bead method. Briefly, 50 μL of buffer was added (25mM Tris, pH8, 100mM NaCl, 1mM EDTA, 10mM MgCl2, 0.01% NP-40, 1 mM DTT, 1mM PMSF, 1mM benzamidine) along with 50 μL of glass beads was mixed with cells for beads beating. Sample was then collected from the beads and 50 μL SDS sample buffer was added, the samples boiled for 3 minutes, and loaded onto a SDS PAGE gel followed by Western blot analysis. To detect the large Sir4-TAP in yeast cell extracts, cell pellets were resuspended in a buffer containing NaOH (1.85M NaOH, 7.4% 2-mercaptoethanol) and incubated on ice for 10 minutes. Next, an equal volume of 20% trichloroacetic acid (TCA) was added, and the samples incubated for 10 minutes on ice. The samples were then centrifuged at top speed for 2 minutes. The pellet was washed in acetone. After drying, the pellet was resuspended in 0.1M NaOH and an equal volume of SDS loading buffer. The samples were resolved on an 8% SDS-PAGE gel and analyzed via Western blot.

**Purification of Sir4-TAP**

Purification of TAP-tagged Sir4 was performed according to published protocols [4,5]. Two liters of cells were grown to OD 2.0 and harvested by centrifugation. Cells were washed in 10% glycerol +1mM PMSF two times. The resulting pellet was resuspended in Sir4-TAP lysis buffer (50 mM HEPES, pH7.6, 100 mM NaCl, 1 mM EDTA, 1 mM EGTA, 5% glycerol, 0.1% NP-40, 50 mM NaF, 10 mM 2-mercaptoethanol, 1mM PMSF) and frozen, drop by drop, in liquid nitrogen. The cells were then broken via grinding in a 6870 Freezer Mill. Following centrifugation, the resulting supernatant was incubated with washed IgG-Sepharose beads (Pharmacia) for 2 hours. After washing, the bound protein was cleaved via TEV protease in TEV cleavage buffer (10mM Tris-HCl, pH 8.0, 100 mM NaCl, 0.5mM EDTA, 0.01% NP-40, 1mM DTT) for 2 hours at 16 ºC. The eluted proteins were diluted using 3 volumes of calmodulin binding buffer (10 mM Tris-HCl, pH8.0, 100 mM NaCl, 1 mM MgAcetate, 1 mM imidazole, 2 mM CaCl_2_, 0.01% NP-40, 5 mM 2-mercaptoethanol) first before used for binding to calmodulin beads for 1 hour at 4 ºC. Following incubation, the beads were washed, and the resulting protein was eluted using calmodulin elution buffer (10 mM Tris-HCl, pH8.0, 100 mM NaCl, 0.01% NP-40, 1 mM MgAcetate, 1 mM imidiazole, 20 mM EGTA, 5 mM 2-mercaptoethanol) and resolved using SDS PAGE. Proteins, as well as ubiquitylated species, were detected by Western blot using antibodies against Sir4, calmodulin binding peptide (CBP), protein A (PAP) and Sir2.

Ubiquitylated Sir4 species were detected using two different methods. First, the above Sir4-TAP purification protocol was followed using strains harboring the pGPD416-Ub plasmid for expression of HA-Ub. Ubiquitylated species were detected using antibodies against the HA epitope. Second, Sir4-TAP was purified as above in cells harboring a plasmid for expression of HA-ub or His-HA-ub (pHis415-HA-ub), and following TEV cleavage, the Sir4-associated ubiquitylated proteins were purified under denatured conditions (8M urea) using Ni-NTA agarose (Qiagen). Ubiquitylated species were detected using antibody against HA.

***In vitro* ubiquitylation assays**

*In vitro* ubiquitylation assays were performed as previously described [6]. Briefly, a reaction mixture with 25 ng of yeast E1, 155 ng E2 (Cdc34), 3 μg of substrate (purified Sir4-CBP from yeast cells), 25 ng of Flag-Ub, 5 μM Ub aldehyde, and increasing amounts of Dia2 E3 ligase were assembled in Ubiquitylation buffer (50 mM Tris-HCl, pH 7.5, 5 mM MgCl2, 0.6 mM DTT, 2 mM ATP, and 200 μM Na_3_VO_4_). Following incubation for 1 hour at 30˚C, Sir4-CBP and associated proteins were recovered using 15 μl of calmodulin beads. Ubiquitylated proteins were detected using antibodies against the Flag epitope. Flag-Ub, yeast E1 and ubiquitin aldehyde were purchased from Boston Biochem. Recombinant Cdc34 was purified from *E. coli*, and Sir4 was purified from yeast cells by tandem affinity purification as described above.

To assay Dia2 activity in cell extracts, cells expressing MYC-Dia2 full length or MYC-Dia2 F-boxΔ were harvested at OD_600_ and washed, and cell pellets were resuspended in 1X volume of Sir4-TAP lysis buffer. Protein was extracted by grinding using the 6870 Freezer Mill. Extracts were cleared using centrifugation. After cell extracts were normalized using Coomassie staining and Western blot with antibodies against the MYC epitope of tagged Dia2, similar amounts of extracts (10-20 μg) from cells expressing full length MYC-Dia2 of MYC-Dia2 F-box∆ were used for ubiquitylation assays described above.

To assay SCF^Dia2^ activity using purified Dia2, cell extracts prepared from MYC-Dia2 full length and F-boxΔ were incubated with 9E10 (c-MYC) antibody for 2 hours at 4˚C. Next, the lysates were incubated with protein A beads (GE Healthcare) for 2 hours at 4˚C. The beads were then washed gently three times with Sir4-TAP lysis buffer. Bound protein was confirmed via Western blot with antibodies against the MYC epitope. The protein bound beads were used for ubiquitylation assays and binding assays.

***In vitro* binding assays**

To test whether Sir4 bound to SCF^Dia2^, we used the MYC-Dia2 complex purified as described above and control protein A beads prepared by incubating beads with the same amount of 9E10 antibody used to purify the MYC-Dia2 complex. *In vitro* translated ^35^S^-^methionine-labeled-Sir4 was prepared using the TNT rapid *in vitro* transcription translation kit (Promega). Equal amounts of beads were incubated with two different amounts of ^35^S-Sir4 overnight in Buffer A100 (24mM Tris, pH7.2, 10% glycerol, 0.01%NP-40, 100mM NaCl and 2mM DTT). Beads were washed 5X with Buffer A and the protein then eluted with SDS sample buffer. Binding reactions were resolved using SDS-PAGE. MYC-Dia2 complex was detected using Western Blot analysis with 9E10 antibody. Bound ^35^S-Sir4 was detected via autoradiography.

**Supporting Information References**

1. Tong AH, Evangelista M, Parsons AB, Xu H, Bader GD, et al. (2001) Systematic genetic analysis with ordered arrays of yeast deletion mutants. Science 294: 2364-2368.

2. Huang S, Zhou H, Katzmann D, Hochstrasser M, Atanasova E, et al. (2005) Rtt106p is a histone chaperone involved in heterochromatin-mediated silencing. Proc Natl Acad Sci U S A 102: 13410-13415.

3. Schmitt ME, Brown TA, Trumpower BL (1990) A rapid and simple method for preparation of RNA from Saccharomyces cerevisiae. Nucleic Acids Res 18: 3091-3092.

4. Burgess RJ, Zhou H, Han J, Zhang Z (2010) A role for Gcn5 in replication-coupled nucleosome assembly. Mol Cell 37: 469-480.

5. Hoppe GJ, Tanny JC, Rudner AD, Gerber SA, Danaie S, et al. (2002) Steps in assembly of silent chromatin in yeast: Sir3-independent binding of a Sir2/Sir4 complex to silencers and role for Sir2-dependent deacetylation. Mol Cell Biol 22: 4167-4180.

6. Han J, Li Q, McCullough L, Kettelkamp C, Formosa T, et al. (2010) Ubiquitylation of FACT by the cullin-E3 ligase Rtt101 connects FACT to DNA replication. Genes Dev 24: 1485-1490.

7. Huang S, Zhou H, Tarara J, Zhang Z (2007) A novel role for histone chaperones CAF-1 and Rtt106p in heterochromatin silencing. Embo J 26: 2274-2283.

8. Thomas BJ, Rothstein R (1989) The genetic control of direct-repeat recombination in Saccharomyces: the effect of rad52 and rad1 on mitotic recombination at GAL10, a transcriptionally regulated gene. Genetics 123: 725-738.

9. Li Q, Zhou H, Wurtele H, Davies B, Horazdovsky B, et al. (2008) Acetylation of histone H3 lysine 56 regulates replication-coupled nucleosome assembly. Cell 134: 244-255.

10. Li Q, Fazly AM, Zhou H, Huang S, Zhang Z, et al. (2009) The elongator complex interacts with PCNA and modulates transcriptional silencing and sensitivity to DNA damage agents. PLoS Genet 5: e1000684.
